# Microbial signals, MyD88, and lymphotoxin drive TNF-independent intestinal epithelial tissue damage

**DOI:** 10.1172/JCI154993

**Published:** 2022-03-01

**Authors:** Iulia Rusu, Elvira Mennillo, Jared L. Bain, Zhongmei Li, Xiaofei Sun, Kimberly M. Ly, Yenny Y. Rosli, Mohammad Naser, Zunqiu Wang, Rommel Advincula, Philip Achacoso, Ling Shao, Bahram Razani, Ophir D. Klein, Alexander Marson, Jessie A. Turnbaugh, Peter J. Turnbaugh, Barbara A. Malynn, Averil Ma, Michael G. Kattah

**Affiliations:** 1 Department of Medicine, UCSF, San Francisco, California, USA.; 2 Gladstone Institutes, San Francisco, California, USA.; 3 Department of Microbiology and Immunology and; 4 Biological Imaging Development CoLab, UCSF, San Francisco, California, USA.; 5 Department of Medicine, University of Southern California, Los Angeles, California, USA.; 6 Department of Dermatology,; 7 Departments of Orofacial Sciences and Pediatrics, Program in Craniofacial Biology, and; 8 Institute for Human Genetics, UCSF, San Francisco, California, USA.; 9 Innovative Genomics Institute, University of California, Berkeley, California, USA.; 10 Parker Institute for Cancer Immunotherapy, San Francisco, California, USA.; 11 Chan Zuckerberg Biohub, San Francisco, California, USA.

**Keywords:** Gastroenterology, Immunology, Apoptosis survival pathways, Inflammatory bowel disease, Mouse models

## Abstract

Anti-TNF antibodies are effective for treating patients with inflammatory bowel disease (IBD), but many patients fail to respond to anti-TNF therapy, highlighting the importance of TNF-independent disease. We previously demonstrated that acute deletion of 2 IBD susceptibility genes, *A20* (*Tnfaip3*) and *Abin-1* (*Tnip1*), in intestinal epithelial cells (IECs) sensitized mice to both TNF-dependent and TNF-independent death. Here we show that TNF-independent IEC death after *A20* and *Abin-1* deletion was rescued by germ-free derivation or deletion of *MyD88*, while deletion of *Trif* provided only partial protection. Combined deletion of *Ripk3* and *Casp8*, which inhibits both apoptotic and necroptotic death, completely protected against death after acute deletion of *A20* and *Abin-1* in IECs. *A20*- and *Abin-1*–deficient IECs were sensitized to TNF-independent, TNFR1-mediated death in response to lymphotoxin α (LTα) homotrimers. Blockade of LTα in vivo reduced weight loss and improved survival when combined with partial deletion of *MyD88*. Biopsies of inflamed colon mucosa from patients with IBD exhibited increased *LTA* and *IL1B* expression, including a subset of patients with active colitis on anti-TNF therapy. These data show that microbial signals, *MyD88*, and LTα all contribute to TNF-independent intestinal injury.

## Graphical abstract

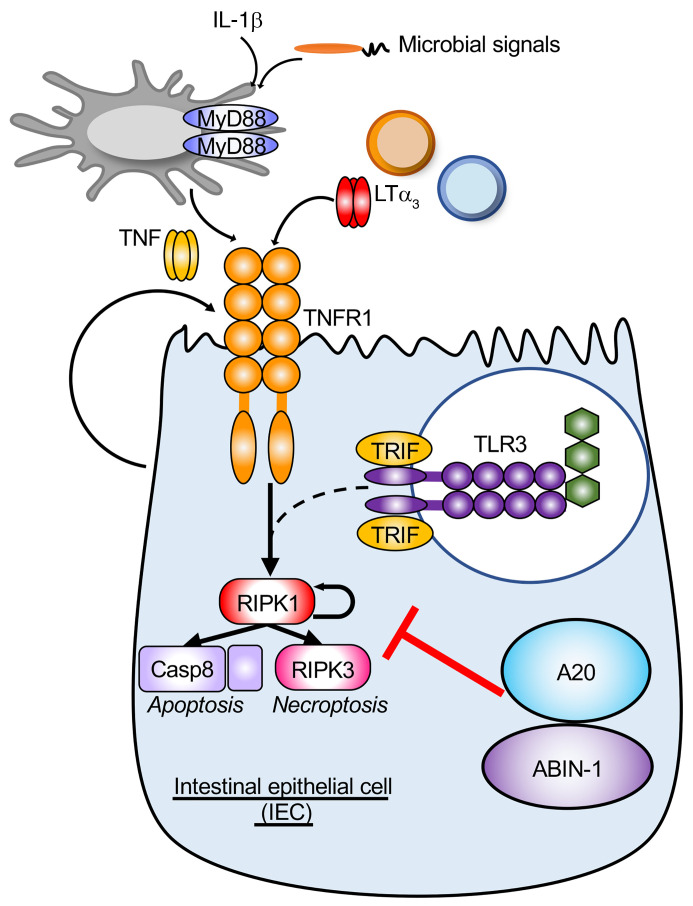

## Introduction

The intestinal epithelium, a single-cell-layer protective lining, is critically important for preventing inflammatory responses to a vast array of microbial stimuli. Inflammatory bowel disease (IBD) is the result of an abnormal immune response to microbial stimuli in genetically susceptible individuals, culminating in intestinal epithelial cell (IEC) injury (1–6). IECs play a central role in IBD pathogenesis, and many candidate IBD-associated genes influence IEC biology, including *ITLN1*, *NOS2,* *ATG16L1*, *XBP1*, *A20* (*TNFAIP3*), *ABIN-1* (*TNIP1*), among others (7–12). Understanding the genetic, microbial, and environmental factors that influence IEC death and injury may enable identification of biomarkers for precision medicine and highlight novel pathways that could be targeted for treating patients with IBD.

Polymorphisms in *A20* (*TNFAIP3*) and *ABIN-1* (*TNIP1*) are linked to a variety of inflammatory disorders affecting multiple tissues, including IBD (13–17). Germline mutations causing *A20* haploinsufficiency have been identified in patients with a systemic inflammatory disorder characterized in part by intestinal ulcerations, typically with pediatric or even infantile onset (18–20). A20 and ABIN-1 are ubiquitin-interacting proteins that interact with each other at the protein level, and both restrict cell death as well as NF-κB signaling downstream of TNF and Toll-like receptors (TLRs) (21–36). A20 and ABIN-1 are both expressed in human and murine intestinal epithelium. Mice with *A20*-deficient IECs develop normally, but are more susceptible to dextran sodium sulfate–induced colitis as well as cancer induced by A20-deficient myeloid cells or collaborating oncogenes (37–39). A20 and ABIN-1 have important roles in restricting inflammation in multiple tissue types, but much remains to be learned about the role of A20 and ABIN-1 specifically in intestinal epithelial tissue damage.

We previously demonstrated that IEC-specific deletion of either *A20* or *Abin-1* alone does not lead to overt weight loss or intestinal injury, but acute simultaneous deletion of both *A20* and *Abin-1* leads to spontaneous IEC apoptosis, fulminant enterocolitis, and rapid mouse lethality (9). In this setting, *A20* and *Abin-1* cooperatively restrict both TNF-dependent and TNF-independent IEC death. TNF-independent IEC death is substantially less well characterized than TNF-dependent death, and can involve microbial signals, *Trif* (*Ticam1*), *Zbp1*, IFN signaling, ripoptosome activation, or other inflammatory death pathways (40–45). Anti-TNF therapy remains one of the most effective approaches for treating Crohn’s disease (CD) and ulcerative colitis (UC), but roughly one-third of patients have no response and one-third of patients lose response over time (46–49). Therefore, understanding the gene products that control TNF-independent IEC injury could have significant translational relevance for anti-TNF nonresponders. To better understand the pathways leading to TNF-independent IEC injury we performed in vivo and in vitro analysis of IECs after acute simultaneous deletion of *A20* and *Abin-1*.

## Results

**Figure 1:**
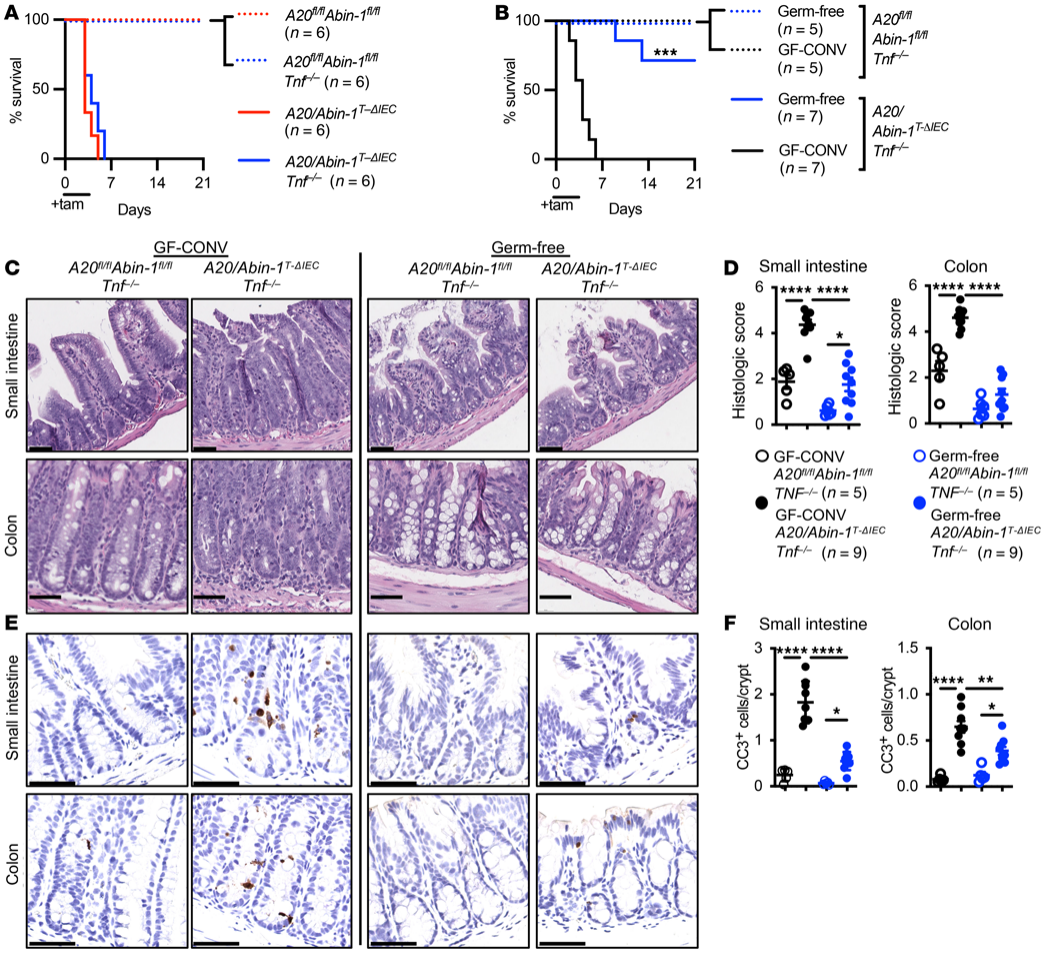
Germ-free *A20/Abin-1*^T-ΔIEC^ *Tnf^–/–^* mice are protected from TNF-independent apoptotic IEC death in vivo. (**A**) Kaplan-Meier survival curves of the indicated genotypes of tamoxifen-treated mice. (**B**) Kaplan-Meier survival curves of tamoxifen-treated mice with the indicated genotypes, either germ-free or conventionalized with cecal contents from SPF mice (GF-CONV). (**C**) Representative H&E images, (**D**) histological scoring, (**E**) representative CC3 IHC images, and (**F**) CC3^+^ cells per crypt of small intestine and colon sections 40 hours after tamoxifen treatment in mice with the indicated genotype; each data point represents 1 mouse (mean ± SEM). The legend for panel **F** is shown in panel **D**. For panels **A** and **B**, statistical significance was assessed by log-rank Mantel-Cox test, comparing *A20/Abin-1*^T-ΔIEC^ to *A20/Abin-1*^T-ΔIEC^ *Tnf^–/–^* mice in panel **A** and germ-free to GF-CONV mice in panel **B**. For panels **D** and **F**, significance was assessed by 1-way ANOVA with Tukey’s multiple-comparison test. Only significant differences are shown. *P < 0.05; **P < 0.01; ***P < 0.001; ****P < 0.0001. Scale bars: 100 μm. Data represent at least 2 independent experiments.

*Germ-free A20/Abin-1*^T-ΔIEC^*Tnf^–/–^ mice are protected from TNF-independent IEC death*. Mice with floxed A20 (*A20^fl/fl^*) and floxed Abin-1 (*Abin-1^fl/fl^*) on a *Vil-cre-ER^T2+^* background (*A20/Abin-1*^T-ΔIEC^) undergo acute deletion of *A20* and *Abin-1* in IECs upon treatment with tamoxifen, culminating in spontaneous apoptotic IEC death, severe enterocolitis, and rapid mouse lethality (9). This death occurs on a *Tnf^+/+^* or *Tnf^–/–^* background, demonstrating the important role of TNF-independent death in this model. Tamoxifen delivery by intraperitoneal (i.p.) oil injection has been reported to cause peritoneal inflammation, foam cell formation, and depletion of resident macrophages (50). To exclude the possibility that sterile peritonitis contributes to TNF-independent death in *A20/Abin-1*^T-ΔIEC^ *Tnf^–/–^* mice, we treated mice with tamoxifen by oral gavage rather than i.p. A higher dose of tamoxifen was required to delete *A20* and *Abin-1* in IECs from the small intestine and colon by oral gavage (Supplemental Figure 1A; supplemental material available online with this article; https://doi.org/10.1172/JCI154993DS1), and with this approach *A20/Abin-1*^T-ΔIEC^ *Tnf^–/–^* mice died with similar kinetics to those of *A20/Abin-1*^T-ΔIEC^ mice (Figure 1A). Enteroids derived from *A20/Abin-1*^T-ΔIEC^ *Tnf^–/–^* mice undergo deletion of *A20* and *Abin-1* when treated with 200 nM 4-hydroxytamoxifen (4-OHT) in vitro, but they are protected from spontaneous cell death (Supplemental Figure 1, B–D). This suggests that IEC-extrinsic factors in vivo drive TNF-independent IEC death and mortality in *A20/Abin-1*^T-ΔIEC^ *Tnf^–/–^* mice. Since in vitro IEC enteroid cultures are sterile, we considered that microbial signals might promote death in vivo. While our prior studies suggested that broad-spectrum-antibiotic treatment was insufficient to rescue *A20/Abin-1*^T-ΔIEC^ *Tnf^–/–^* mice (9), we hypothesized that residual microbes in these mice could trigger IEC death. Accordingly, we derived *A20/Abin-1*^T-ΔIEC^ *Tnf^–/–^* germ-free mice by cesarean section. Germ-free *Tnf^–/–^* mice were largely protected from death upon deletion of *A20* and *Abin-1* in IECs (Figure 1B). To control for developmental alterations by germ-free derivation, we conventionalized germ-free *A20/Abin-1*^T-ΔIEC^ *Tnf^–/–^* mice with cecal contents from corresponding specific pathogen–free (SPF) mice in our facility. Germ-free mice conventionalized with cecal contents from SPF *A20/Abin-1*^T-ΔIEC^ *Tnf^–/–^* mice (GF-CONV) exhibited rapid mortality upon deletion of *A20* and *Abin-1* (Figure 1B), suggesting the increased survival of germ-free mice was not due to a developmental aberration. Therefore, microbial signals contribute to TNF-independent IEC death in the setting of acute *A20* and *Abin-1* deletion.

Although germ-free *A20/Abin-1*^T-ΔIEC^ *Tnf^–/–^* mice exhibited increased survival, it was unclear whether this was due to reduced IEC death or merely due to broadly reduced septic sequelae under germ-free conditions. Histologically, acute deletion of *A20* and *Abin-1* in the intestinal epithelium caused rapid intestinal epithelial denudation, inflammatory infiltrate, cryptitis, and loss of mucosal architecture in both the small intestine and colon within 40 hours in GF-CONV mice (Figure 1, C and D). In contrast, germ-free *A20/Abin-1*^T-ΔIEC^ *Tnf^–/–^* mice exhibited far less histologic injury (Figure 1, C and D). Since IEC loss in this model is further characterized by massive apoptotic IEC death, we performed cleaved CASP3 (CC3) immunohistochemistry. In parallel to the reduction in histologic disease severity, we observed dramatically reduced CC3 in IECs of germ-free *A20/Abin-1*^T-ΔIEC^ *Tnf^–/–^* mice as compared with GF-CONV counterparts (Figure 1, E and F).

These results highlight that *A20/Abin-1*^T-ΔIEC^ *Tnf^–/–^* mice provide a window into studying TNF-independent IEC death. *A20/Abin-1*^T-ΔIEC^ *Tnf^–/–^* mice die due to spontaneous fulminant IEC death in vivo, but those IECs survive in vitro in the absence of hematopoietic cells, autocrine TNF, and microbial stimuli. When microbial stimuli are removed under germ-free conditions, the *A20*- and *Abin-1*–deficient IECs survive in vivo even with hematopoietic cells and other potential cytotoxic factors present. Interestingly, some germ-free *A20/Abin-1*^T-ΔIEC^ *Tnf^–/–^* mice die (Figure 1B), suggesting that there may be some sterile inflammatory factors that can contribute to TNF-independent IEC death, but microbial factors are a primary driver of intestinal inflammation in this model.

**Figure 2:**
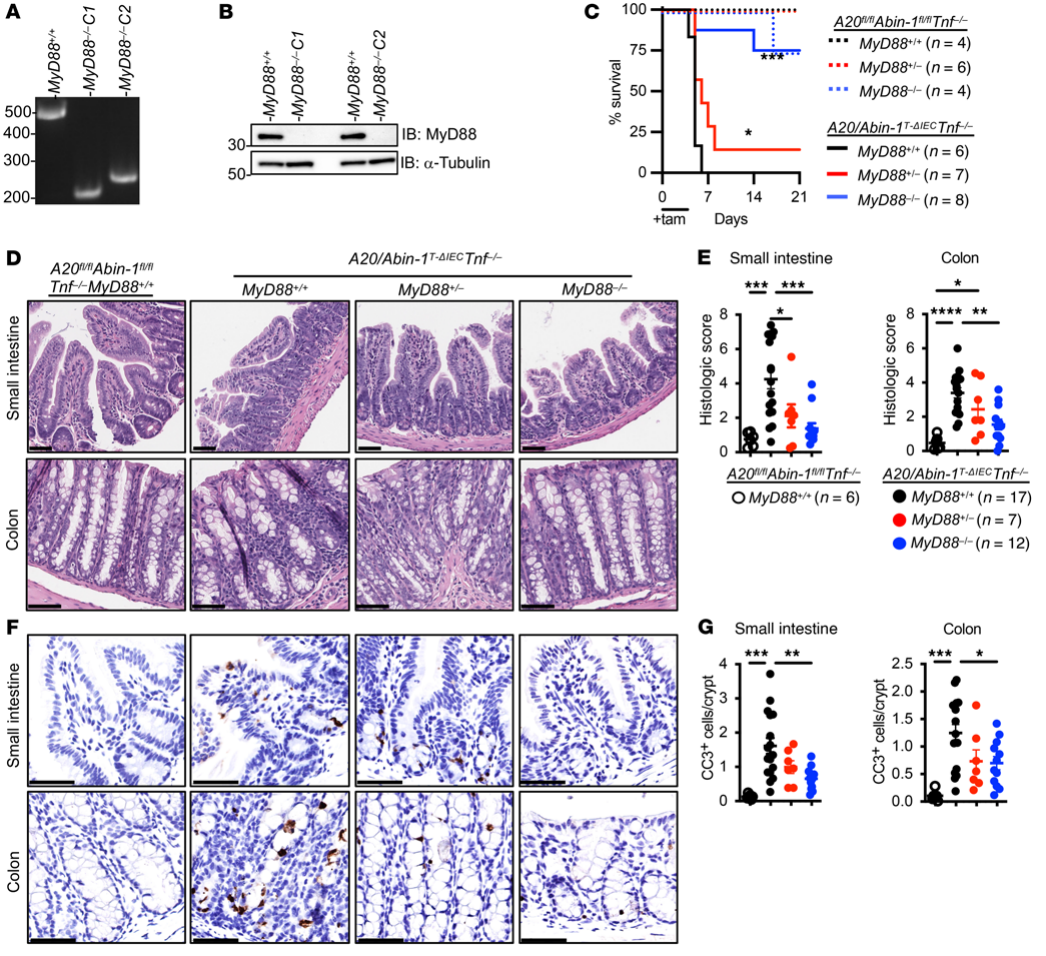
Deletion of *MyD88* significantly improves survival, reduces epithelial injury, and decreases IEC apoptosis in *A20/Abin-1*^T-ΔIEC^ *Tnf^–/–^* mice. (**A**) Agarose gel electrophoresis and (**B**) immunoblot of splenocyte lysates from *A20/Abin-1*^T-ΔIEC^ *Tnf^–/–^* mice with the indicated *MyD88* genotypes. (**C**) Kaplan-Meier survival curves of the indicated genotypes of tamoxifen-treated mice. (**D**) Representative H&E images, (**E**) histological scoring, (**F**) representative CC3 IHC images, and (**G**) CC3^+^ cells per crypt of small intestine and colon sections 40 hours after tamoxifen treatment in mice with the indicated genotype; each data point represents 1 mouse (mean ± SEM). The legend for panel **G** is shown in panel **E**. For panel **C**, statistical significance comparing *A20/Abin-1*^T-ΔIEC^ *Tnf^–/–^MyD88*^+/+^ mice to *MyD88*^–/–^ and *MyD88*^+/–^ mice was assessed by log-rank Mantel-Cox test. For panels **E** and **G**, statistical significance was assessed by 1-way ANOVA with Tukey’s multiple-comparison test. Only significant differences are shown. *P < 0.05; **P < 0.01; ***P < 0.001; ****P < 0.0001. Scale bars: 100 μm. Data represent at least 2 independent experiments.

*Deletion of MyD88, and to a lesser extent Trif, rescues A20/Abin-1*^T-ΔIEC^*Tnf^–/–^ mice*. Given the dramatic improvement in survival and intestinal epithelial integrity in germ-free *A20/Abin-1*^T-ΔIEC^*Tnf^–/–^* mice, we hypothesized that microbial signaling through *MyD88* mediated the intestinal inflammation in this model. To facilitate combining of multiple mutant alleles, we targeted *MyD88* in *A20/Abin-1*^T-ΔIEC^ *Tnf^–/–^* zygotes as previously described (51). Using 2 guide RNAs (gRNAs) targeting exon 1 of *MyD88,* we generated 2 founder strains of mice with deletions at the *MyD88* locus, *A20/Abin-1*^T-ΔIEC^ *Tnf^–/–^* *MyD88^–/–^ C1* and *C2* (Figure 2A and Supplemental Figure 2, A and B). We confirmed deletion of MyD88 at the protein level in both mouse strains (Figure 2B). *A20/Abin-1*^T-ΔIEC^ *Tnf^–/–^* *MyD88^–/–^ C1* and *C2* mice behaved identically, and so are presented in aggregate for clarity. Heterozygous deletion of *MyD88* conferred a modest improvement in survival, while complete deletion of *MyD88* led to a marked improvement in survival in *A20/Abin-1*^T-ΔIEC^ *Tnf^–/–^* mice (Figure 2C). The histologic phenotype paralleled the survival benefit, where the intestinal epithelium from *A20/Abin-1*^T-ΔIEC^ *Tnf^–/–^* *MyD88^–/–^* mice exhibited significantly less inflammatory injury in the small intestine and colon as compared with *A20/Abin-1*^T-ΔIEC^ *Tnf^–/–^* mice (Figure 2, D and E). Similarly, deletion of *MyD88* significantly reduced the frequency of apoptotic CC3^+^ IECs as compared with their *MyD88*^+/+^ counterparts (Figure 2, F and G). *A20/Abin-1*^T-ΔIEC^ *Tnf^–/–^* *MyD88^+/–^* mice exhibited intermediate histologic injury and CC3 frequency (Figure 2, D–G). These results are surprising given that *MyD88* plays a critical role in intestinal homeostasis, and its deletion has been reported to increase susceptibility to other mouse models of colitis (52–54).

**Figure 3:**
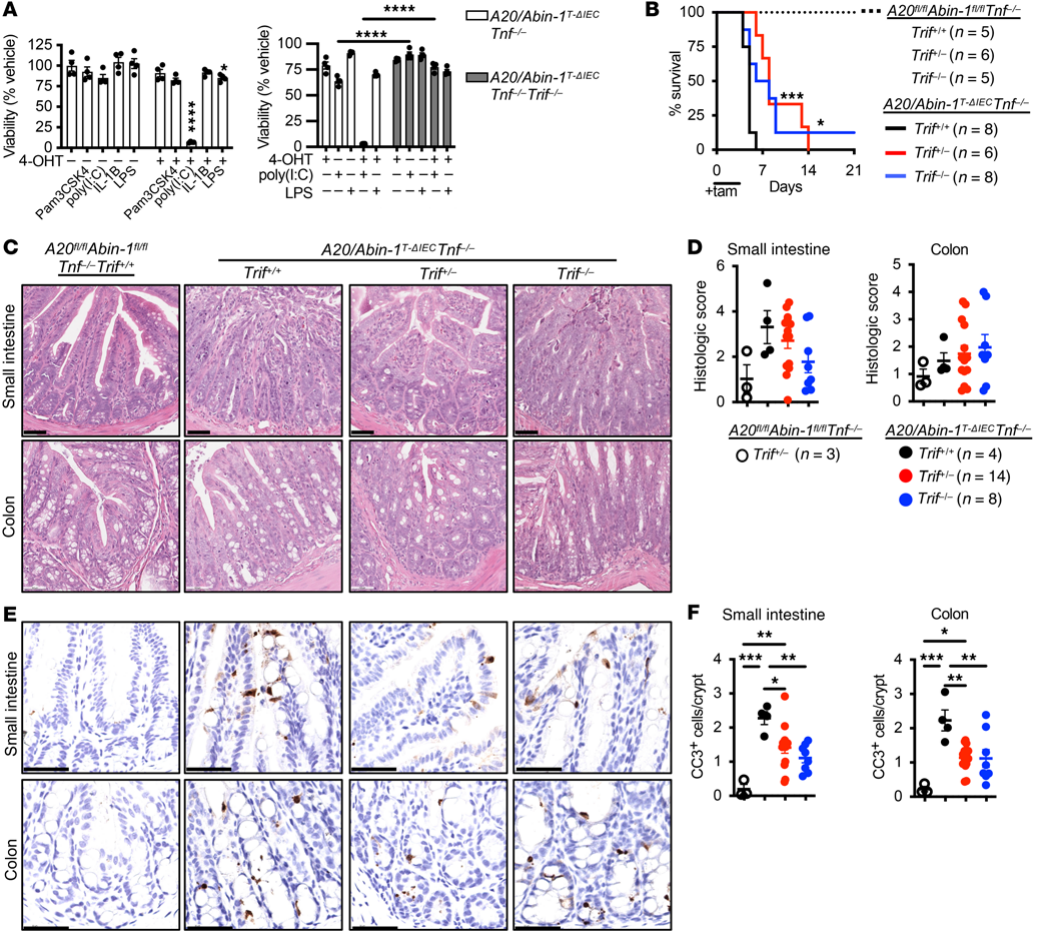
Deletion of *Trif* partially improves survival and modestly reduces IEC apoptosis in *A20/Abin-1*^T-ΔIEC^ *Tnf^–/–^* mice. (**A**) Quantitative luminescent cell viability assay of enteroids with the indicated genotype treated with 4-OHT or vehicle for 24 hours and then treated with the indicated stimuli for 24 hours [mean ± SEM; 500 ng/mL Pam3CSK4, 25 μg/mL poly(I:C), 25 ng/mL IL-1β, 500 ng/mL LPS]. (**B**) Kaplan-Meier survival curves of the indicated genotypes of tamoxifen-treated mice. (**C**) Representative H&E images, (**D**) histological scoring, (**E**) representative CC3 IHC images, and (**F**) CC3^+^ cells per crypt of small intestine and colon sections 40 hours after tamoxifen treatment in mice with the indicated genotype; each data point represents 1 mouse (mean ± SEM). The legend for panel **F** is shown in panel **D**. For panel **A**, statistical significance was assessed using 2-way ANOVA with Bonferroni’s multiple-comparison test. For panel **B**, significance comparing *A20/Abin-1*^T-ΔIEC^ *Tnf^–/–^* *Trif^+/+^* mice to *Trif^–/–^* and *Trif^+/–^* mice was assessed by log-rank Mantel-Cox test. For panels **D** and **F**, statistical significance was assessed by 1-way ANOVA with Tukey’s multiple-comparison test. Only significant differences are shown. *P < 0.05; **P < 0.01; ***P < 0.001; ****P < 0.0001. Scale bars: 100 μm. Data represent at least 2 independent experiments.

*MyD88* expression in IECs maintains intestinal epithelial integrity and homeostasis (53, 54). Given the improved survival of *A20/Abin-1*^T-ΔIEC^ *Tnf^–/–^* *MyD88^–/–^* mice, we next examined whether MyD88 activation directly induces apoptotic IEC death using *A20/Abin-1*^T-ΔIEC^ *Tnf^–/–^* primary small intestinal enteroid cultures. MyD88 mediates signaling downstream of TLRs and IL-1 family members (55, 56), so we stimulated IECs with Pam3CSK4 (a TLR2/1 agonist). This ligand did not induce significant death in *A20/Abin-1*^T-ΔIEC^ *Tnf^–/–^* enteroids (Figure 3A). Similarly, IL-1β and IL-18, two IL-1 family members that activate MyD88, did not directly induce death in *A20/Abin-1*^T-ΔIEC^ *Tnf^–/–^* enteroids (Figure 3A and Supplemental Figure 3A). Although *MyD88* deletion rescues *A20/Abin-1*^T-ΔIEC^ *Tnf^–/–^* mice, MyD88 activation was not sufficient for IEC death in vitro.

TRIF contains a RIP homotypic interaction motif (RHIM) domain and interacts with receptor-interacting serine/threonine kinase 3 (RIPK3) to induce death signaling downstream of TLR3 in response to the dsRNA analog polyinosinic-polycytidylic acid [poly(I:C)] (43, 57–60). We tested the susceptibility of *A20/Abin-1*^T-ΔIEC^ *Tnf^–/–^* enteroids to poly(I:C)-induced death, and we observed that deletion of *A20* and *Abin-1* in IECs dramatically increased susceptibility to poly(I:C)-induced death (Figure 3A). LPS, in contrast, induced minimal cytotoxicity (Figure 3A). Both poly(I:C) and LPS activate TRIF, but IEC death in response to LPS has previously been reported to depend on TNF, in contrast to poly(I:C) (43). We deleted *Trif* in *A20/Abin-1*^T-ΔIEC^ *Tnf^–/–^* mice using CRISPR/Cas9 editing and generated a strain of mice with a deletion and premature stop codon at the *Trif* locus (Supplemental Figure 3, B and C). Splenocytes derived from *A20/Abin-1*^T-ΔIEC^ *Tnf^–/–^* *Trif^–/–^* mice exhibited significantly reduced IFN-β production in response to poly(I:C), confirming functional deletion at the protein level (Supplemental Figure 3D). To determine if *Trif* deletion protected *A20*- and *Abin-1*–deficient IECs from TNF-independent death in vitro, we expanded enteroid cultures from these mice. *A20/Abin-1*^T-ΔIEC^ *Tnf^–/–^* *Trif^–/–^* enteroids were almost entirely resistant to poly(I:C)- and LPS-induced cell death (Figure 3A). Therefore, A20 and ABIN-1 cooperatively restrict TRIF-mediated death in response to TLR3 and TLR4 agonists in vitro.

Given that *Trif* deletion rescued TNF-independent death in response to poly(I:C) and LPS in vitro, we treated *A20/Abin-1*^T-ΔIEC^ *Tnf^–/–^* *Trif^–/–^* mice with tamoxifen to see if *Trif* deletion also protected *A20*- and *Abin-1*–deficient IECs in vivo. Deletion of *Trif* in *A20/Abin-1*^T-ΔIEC^ *Tnf^–/–^* mice provided a modest survival benefit, with slightly delayed median survival relative to *Trif^+/+^* controls (Figure 3B). Interestingly, heterozygous and homozygous deletion of *Trif* conferred similar survival benefit. We examined the small intestine and colon 40 hours after tamoxifen-induced deletion of *A20* and *Abin-1* in *A20/Abin-1*^T-ΔIEC^ *Tnf^–/–^* *Trif^–/–^* mice and saw a trend toward improvement in histologic severity, but this was not statistically significant when compared to the *A20/Abin-1*^T-ΔIEC^ *Tnf^–/–^* mice (Figure 3, C and D). Deletion of *Trif* partially reduced CC3 frequency, but not enough to prevent lethality in the *A20/Abin-1*^T-ΔIEC^ *Tnf^–/–^* mice (Figure 3, E and F). While poly(I:C) potently induces *Trif*-dependent cytotoxicity in *A20*- and *Abin-1*–deficient IECs in vitro, deletion of *Trif* does not protect against TNF-independent intestinal injury in vivo.

**Figure 4:**
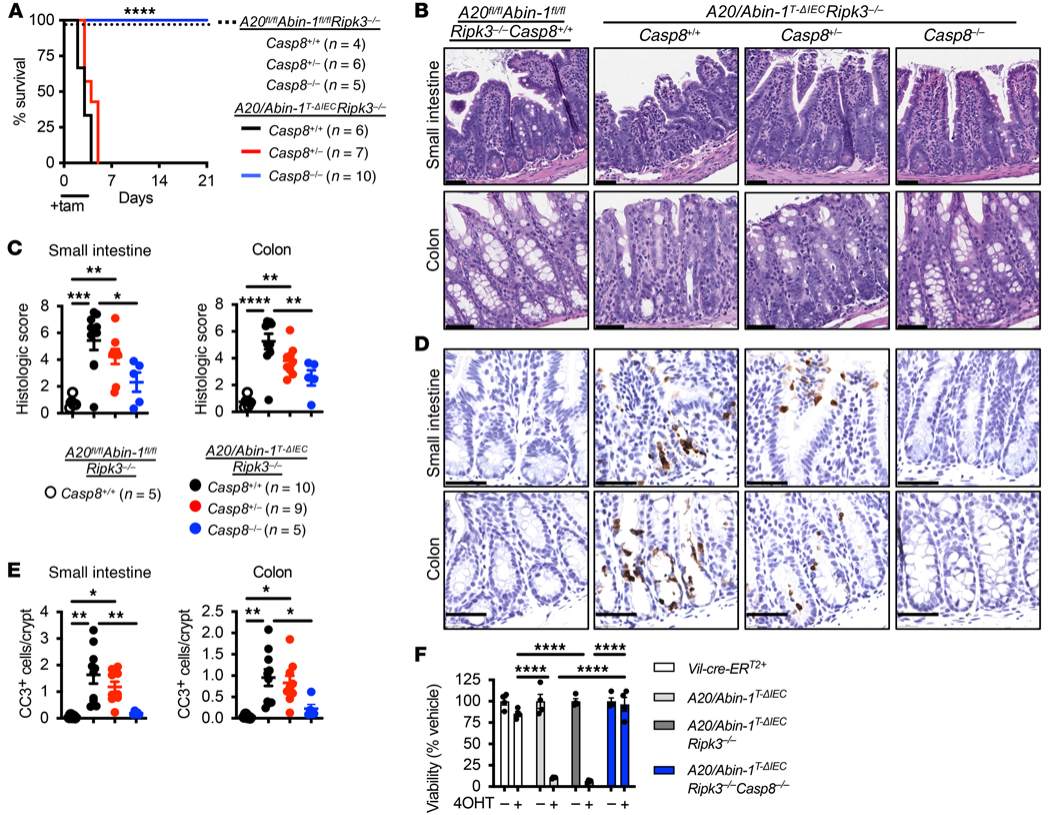
Combined deletion of *Ripk3* and *Casp8* completely protects against death, epithelial injury, and IEC apoptosis in *A20/Abin-1*^T-ΔIEC^ mice. (**A**) Kaplan-Meier survival curves of the indicated genotypes of tamoxifen-treated mice. (**B**) Representative H&E images, (**C**) histological scoring, (**D**) representative CC3 IHC images, and (**E**) CC3^+^ cells per crypt of small intestine and colon sections 40 hours after tamoxifen treatment in mice with the indicated genotype; each data point represents 1 mouse (mean ± SEM). The legend for panel **E** is shown in panel **C**. (**F**) Quantitative luminescent cell viability assay of enteroids with the indicated genotype treated with 4-OHT for 48 hours (mean ± SEM). For panel **A**, statistical significance comparing *A20/Abin-1*^T-ΔIEC^ *Ripk3^–/–^* *Casp8*^+/+^ mice to *Casp8*^+/–^ and *Casp8*^–/–^ mice was assessed by log-rank Mantel-Cox test. For panels **C**, **E**, and **F**, statistical significance was assessed by 1-way ANOVA with Tukey’s multiple-comparison test. Only significant differences are shown. *P < 0.05; **P < 0.01; ***P < 0.001; ****P < 0.0001. Scale bars: 100 μm. Data represent at least 2 independent experiments.

*Combined deletion of Ripk3 and Casp8 completely protects A20/Abin-1*^T-ΔIEC^*IECs from death*. Deletion of *Trif* did not reverse apoptotic IEC death in *A20/Abin-1*^T-ΔIEC^ *Tnf^–/–^* mice, but A20 is known to inhibit necroptosis as well as apoptosis (23, 61), and combined deletion of *A20* and *Abin-1* sensitizes IECs to both CASP8-dependent apoptotic death and RIPK3-dependent necroptotic death in vitro (9). Deletion of *Ripk3* does not prevent IEC injury after acute deletion of *A20* and *Abin-1*, so we hypothesized that combined deletion of *Ripk3* and *Casp8* would rescue *A20/Abin-1*^T-ΔIEC^ mice. We performed CRISPR/Cas9 editing of the *Casp8* locus in *A20/Abin-1*^T-ΔIEC^ *Ripk3^–/–^* mice. This yielded a strain of mice with a premature stop codon in exon 2 of *Casp8* (Supplemental Figure 4A), with deletion of CASP8 at the protein level confirmed by immunoblotting (Supplemental Figure 4B). *A20/Abin-1*^T-ΔIEC^ *Ripk3^–/–^* mice exhibited 100% lethality, but *A20/Abin-1*^T-ΔIEC^ *Ripk3^–/–^* *Casp8^–/–^* mice were completely protected from death after treatment with tamoxifen (Figure 4A). Histologically, *A20/Abin-1*^T-ΔIEC^ *Ripk3^–/–^* *Casp8^–/–^* mice exhibited markedly improved histologic disease severity (Figure 4, B and C) and CC3 frequency was reduced to near-background levels (Figure 4, D and E). In vitro, enteroids derived from *A20/Abin-1*^T-ΔIEC^ *Ripk3^–/–^* *Casp8^–/–^* mice were protected from both spontaneous IEC death (Figure 4F) and TRIF-mediated cell death (Supplemental Figure 4C). These data collectively demonstrate that simultaneous suppression of both CASP8-dependent apoptosis and RIPK3-dependent necroptosis completely preserves IEC survival after acute deletion of *A20* and *Abin-1* in vitro and in vivo.

**Figure 5:**
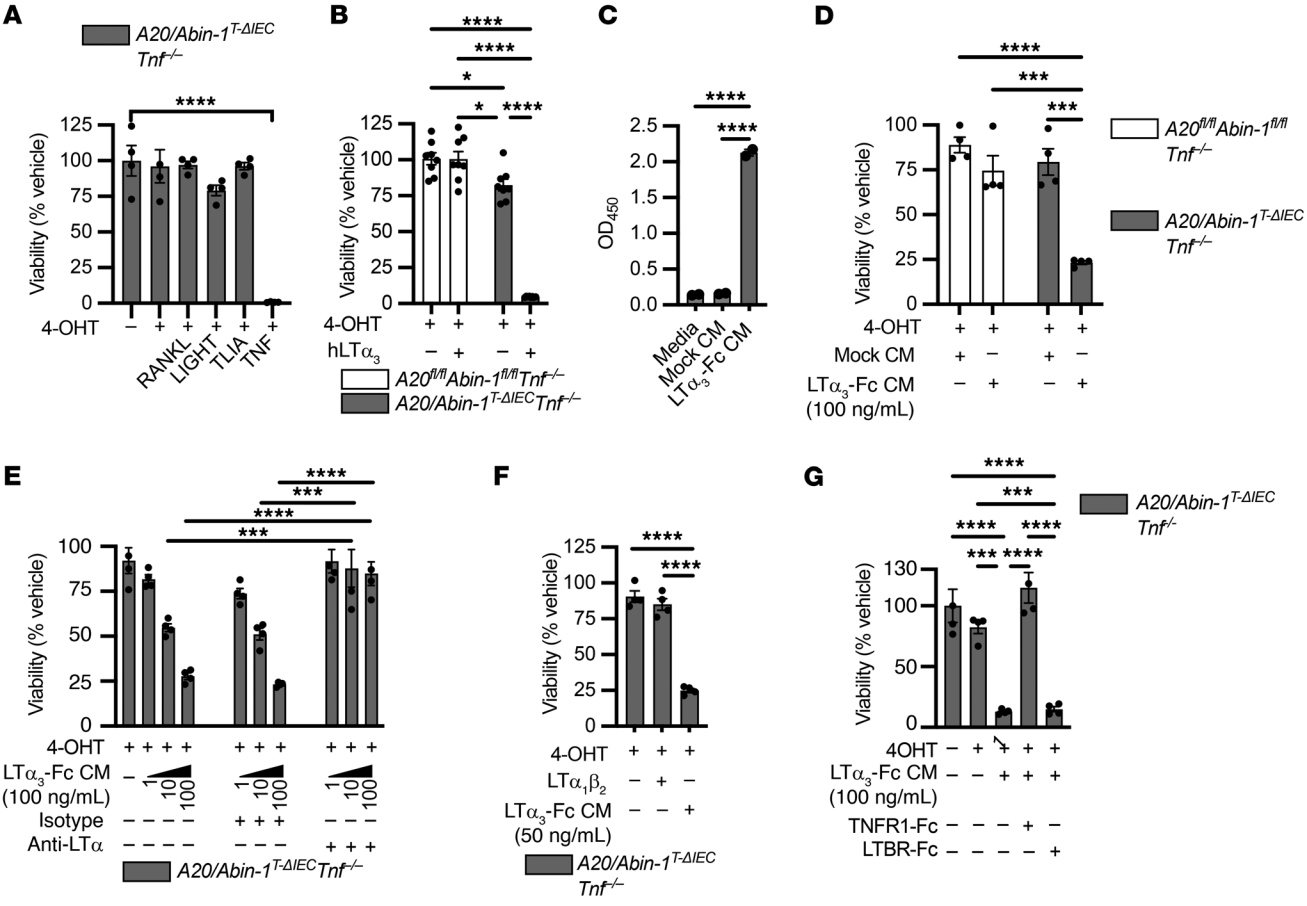
LTα_3_ induces death in *A20/Abin-1*^T-ΔIEC^ *Tnf^–/–^* enteroids through activation of TNFR1. (**A**, **B**, and **D**–**G**) Quantitative luminescent cell viability assay of enteroids with the indicated genotype treated with 4-OHT or vehicle for 24 hours and then treated with the indicated stimuli for 24 hours (mean ± SEM; 25 ng/mL RANKL, LIGHT, TL1A, or TNF; 5 ng/mL recombinant human LTα_3_ [hLTα_3_]; 50 ng/mL LTα_1_β_2_; 10 μg/mL anti-LTα and isotype control; 100 μg/mL TNFR1-Fc, LTBR-Fc). (**C**) ELISA of undiluted conditioned media (CM) from 293T cells either mock transduced (Mock CM) or stably transduced with a mouse LTα_3_-Fc expression construct (LTα_3_-Fc CM) with anti-mIgG2a capture and anti-LTα detection antibodies (mean ± SEM). For panel **A**, significance was assessed by 1-way ANOVA with Dunnett’s multiple-comparison test relative to vehicle. For panels **B** and **E**, significance was assessed using 2-way ANOVA with Tukey’s multiple-comparison test. For panels **C**, **D**, **F**, and **G**, significance was assessed using 1-way ANOVA with Tukey’s multiple-comparison test. Only significant differences are shown. *P < 0.05; ***P < 0.001; ****P < 0.0001. Data represent at least 2 independent experiments.

*LTα_3_ induces apoptosis and necroptosis downstream of TNFR1 in A20/Abin-1*^T-ΔIEC^*Tnf^–/–^ enteroids*. As combined deletion of *Ripk3* and *Casp8* completely prevented IEC death after deletion of *A20* and *Abin-1*, but *Trif* deletion did not provide equivalent survival benefit, we hypothesized that another TNF superfamily member may be contributing to TNF-independent, IEC-extrinsic death in vivo. We previously reported that the TNF superfamily members TWEAK (TNF-like weak inducer of apoptosis), Fas ligand, and TRAIL (TNF-related apoptosis-inducing ligand) failed to induce significant death of *A20/Abin-1*^T-ΔIEC^ *Tnf^–/–^* enteroids in vitro (9). Here we tested RANKL (receptor activator of NF-κΒ ligand), LIGHT (homologous to lymphotoxin, exhibits inducible expression and competes with HSV glycoprotein D for binding to herpesvirus entry mediator, a receptor expressed on T lymphocytes), and TL1A (TNF-like cytokine 1A), none of which induced significant IEC death (Figure 5A).

Although none of those alternative TNF superfamily ligands induced death in *A20/Abin-1*^T-ΔIEC^ *Tnf^–/–^* enteroids, we previously showed that deletion of *A20* and *Abin-1* increases death sensitivity of TNF receptor 1 (TNFR1) to TNF by 1000-fold in IECs (9). Given that TNFR1 sensitivity is significantly increased in *A20*- and *Abin-1*–deficient IECs, we considered alternative TNFR1 ligands. Lymphotoxin α homotrimers (LTα_3_) are reported to bind to TNFR1 and activate death signaling (62–64). We tested human LTα_3_ and observed significant increased susceptibility to death in *A20*- and *Abin-1*–deficient IECs (Figure 5B). To test mouse LTα_3_, we generated a cell line constitutively expressing a mouse LTα_3_-Fc fusion protein and validated the presence of soluble fusion protein (Figure 5C). LTα_3_-Fc fusion protein–conditioned media (LTα_3_-Fc CM) induced significant death in *A20/Abin-1*^T-ΔIEC^ *Tnf^–/–^* enteroids (Figure 5D). Since LTα_3_-Fc CM could contain other factors that drive IEC death in vitro, we wanted to determine whether an anti-LTα monoclonal antibody reversed this activity in vitro. An LTα-specific monoclonal antibody was previously developed and reported to inhibit collagen-induced arthritis (65). We generated purified monoclonal antibody from this hybridoma and observed complete prevention of *A20/Abin-1*^T-ΔIEC^ *Tnf^–/–^* IEC death in response to LTα_3_-Fc CM in vitro (Figure 5E). As a negative control, an isotype control antibody did not block cytotoxic activity (Figure 5E). Subsequently, we wanted to determine whether TNF-independent IEC death in *A20*- and *Abin-1*–deficient IECs in response to LTα_3_-Fc CM was indeed mediated by TNFR1. We added recombinant mouse LTα_1_β_2_, which binds to the LTβ receptor (LTBR) but not TNFR1 (63), and we did not observe any cytotoxicity (Figure 5F). This argues that deletion of *A20* and *Abin-1* in IECs does not increase susceptibility to LTBR-mediated death. We added recombinant mouse LTBR-Fc and TNFR1-Fc fusion proteins and observed that only TNFR1-Fc completely protected *A20/Abin-1*^T-ΔIEC^ *Tnf^–/–^* enteroids from death (Figure 5G). These data demonstrate that deletion of *A20* and *Abin-1* in IECs unveils a profound sensitivity to LTα_3_-induced death downstream of TNFR1. It is important to highlight that the death assays we performed used primary, nonimmortalized IECs in the absence of any death-sensitizing agents (e.g., cycloheximide), unlike most prior studies investigating TNF- and LTα_3_-induced death (62, 64).

**Figure 6:**
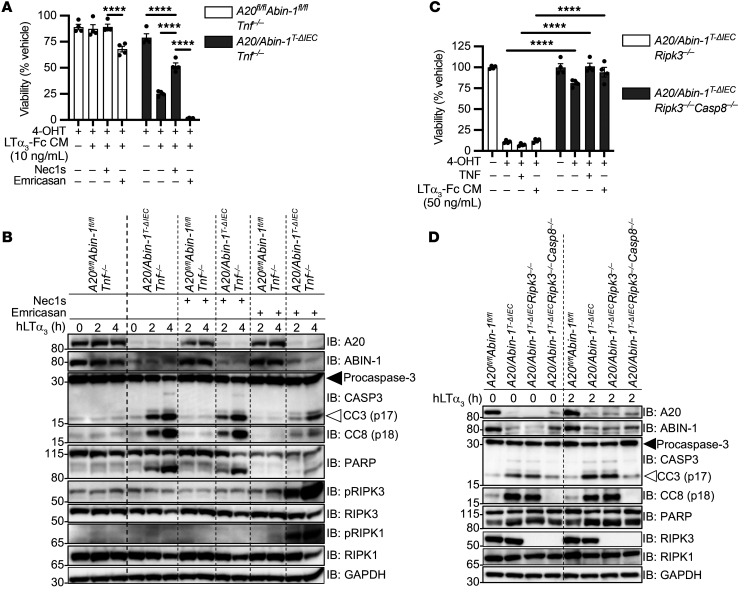
LTα_3_ can induce both CASP8-dependent apoptosis and RIPK3-dependent necroptosis in *A20/Abin-1*^T-ΔIEC^ *Tnf^–/–^* enteroids. (**A** and **C**) Quantitative luminescent cell viability assay of enteroids with the indicated genotypes treated with vehicle, 4-OHT, Nec1s, or emricasan for 24 hours as indicated, and then treated with the indicated stimuli for 24 hours (mean ± SEM; 50 μM Nec1s, emricasan; 50 ng/mL TNF). (**B** and **D**) Immunoblot analyses of enteroid cultures with the indicated genotypes treated with 4-OHT for 22.5 hours, and then vehicle, Nec1s, or emricasan for 1.5 hours, followed by 20 ng/mL recombinant human LTα_3_ (hLTα_3_) as indicated. Lysates were immunoblotted with the antibodies indicated on the right. Solid arrows indicate full-length protein; open arrows indicate cleaved protein. For panel **A**, significance was assessed using 2-way ANOVA with Dunnett’s multiple-comparison test relative to LTα_3_-Fc CM plus Nec1s. For panel **C**, significance was assessed by 2-way ANOVA with Bonferroni’s multiple-comparison test comparing between genotypes for each stimulation condition. Only significant differences are shown. ****P < 0.0001. Data represent at least 2 independent experiments.

Having determined that A20- and ABIN-1–deficient IECs are sensitized to LTα_3_-induced death downstream of TNFR1, we wanted to ascertain whether LTα_3_-induced death was primarily apoptotic or necroptotic. RIPK1 binds CASP8 and RIPK3, and RIPK1’s kinase activity can support both apoptotic and necroptotic death (42, 44, 66, 67). Necrostatin-1s (Nec1s) is a small-molecule RIPK1 kinase inhibitor that suppresses necroptosis and partially suppresses apoptosis (9, 68). As expected, the addition of Nec1s partially prevented LTα_3_-induced death in *A20/Abin-1*^T-ΔIEC^ *Tnf^–/–^* enteroids (Figure 6A). Caspase inhibition increases RIPK3-dependent necroptosis (69–71). Addition of emricasan, a pharmacologic pan-caspase inhibitor, caused increased death in response to LTα_3_, indicating that *A20/Abin-1*^T-ΔIEC^ *Tnf^–/–^* enteroids are sensitized to LTα_3_-induced necroptosis. In agreement with these in vitro death assays, deletion of *A20* and *Abin-1* in *A20/Abin-1*^T-ΔIEC^ *Tnf^–/–^* enteroids leads to significantly increased CC3, cleaved CASP8 (CC8), and cleaved PARP in response to LTα_3_ as compared with control *A20^fl/fl^* *Abin-1^fl/fl^* *Tnf^–/–^* enteroids (Figure 6B). There was no significant increase in phosphorylated RIPK3 (p-RIPK3) (Figure 6B). This pattern is consistent with apoptosis as the primary mode of death in *A20/Abin-1*^T-ΔIEC^ *Tnf^–/–^* enteroids in response to LTα_3_. The addition of Nec1s partially reduced CC3 and CC8 (Figure 6B), which is consistent with partial inhibition of apoptosis in response to RIPK1 kinase activity inhibition. The addition of emricasan reduced CC3 and CC8 but markedly increased p-RIPK1 and p-RIPK3, consistent with increased LTα_3_-induced necroptotic death when caspase activity is inhibited (Figure 6B). These data demonstrate that combined deletion of *A20* and *Abin-1* sensitizes IECs to both TNF-independent apoptosis and necroptosis in response to LTα_3_, although apoptosis is the dominant death pathway.

To further examine necroptosis and apoptosis, we stimulated enteroids derived from *A20/Abin-1*^T-ΔIEC^ *Ripk3^–/–^* and *A20/Abin-1*^T-ΔIEC^ *Ripk3^–/–^* *Casp8^–/–^* mice with TNF and LTα_3_. The addition of exogenous TNF or LTα_3_ did not induce any additional cytotoxicity in *A20/Abin-1*^T-ΔIEC^ *Ripk3^–/–^* *Casp8^–/–^* enteroids (Figure 6C). These in vitro death assays demonstrate that simultaneous blockade of RIPK3-dependent necroptosis and CASP8-dependent apoptosis is required to rescue A20- and ABIN-1–deficient IECs from death in response to TNF or LTα_3_. We next examined death signaling in *A20/Abin-1*^T-ΔIEC^ *Ripk3^–/–^* and *A20/Abin-1*^T-ΔIEC^ *Ripk3^–/–^* *Casp8^–/–^* enteroids. Consistent with our prior studies, *A20/Abin-1*^T-ΔIEC^ *Ripk3^–/–^* enteroids demonstrated increased spontaneous CC3 and CC8 upon deletion of *A20* and *Abin-1*, as compared with *A20^fl/fl^* *Abin-1^fl/fl^* enteroids (Figure 6D). *A20/Abin-1*^T-ΔIEC^ *Ripk3^–/–^* *Casp8^–/–^* enteroids, in contrast, did not exhibit CC3, CC8, or cleaved PARP relative to *A20^fl/fl^* *Abin-1^fl/fl^* enteroids, even in the presence of exogenous LTα_3_ (Figure 6D). Therefore, combined deletion of RIPK3-dependent necroptosis and CASP8-dependent apoptosis completely protects *A20/Abin-1*^T-ΔIEC^ IECs from TNF- or LTα_3_-induced apoptotic and necroptotic death downstream of TNFR1.

**Figure 7:**
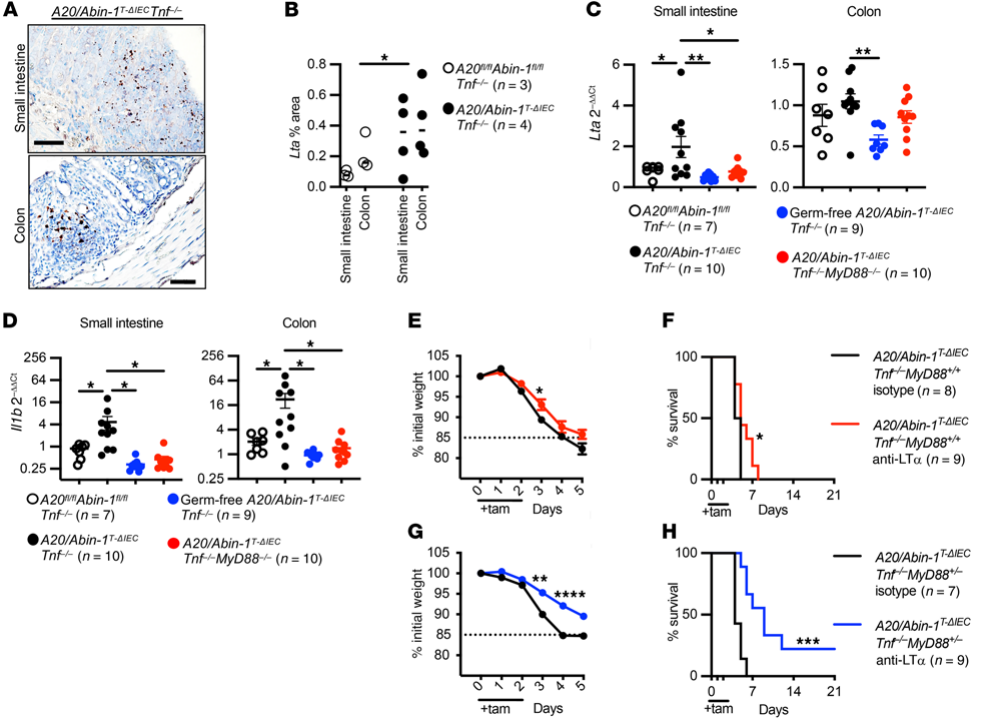
Intestinal *Lta* and *Il1b* are increased in *A20/Abin-1*^T-ΔIEC^ *Tnf^–/–^* mice and anti-LTα improves survival in *A20/Abin-1*^T-ΔIEC^ *Tnf^–/–^* *MyD88^+/+^* and *A20/Abin-1*^T-ΔIEC^ *Tnf^–/–^* *MyD88^+/–^* mice. (**A**) Representative chromogenic RNA-ISH images of *Lta* and (**B**) quantitation of *Lta* area. (**C** and **D**) qPCR for (**C**) *Lta* and (**D**) *Il1b* in small intestine and colon 40 hours after tamoxifen treatment in mice with the indicated genotype; each data point represents 1 mouse (mean ± SEM). (**E** and **G**) Weight curve and (**F** and **H**) Kaplan-Meier survival curves of the indicated genotypes of tamoxifen-treated mice treated with anti-LTα or isotype control antibody. For panel **B**, statistical significance was assessed using 2-way ANOVA. For panels **C** and **D**, significance was assessed using 1-way ANOVA with Dunnett’s multiple-comparison test relative to *A20/Abin-1*^T-ΔIEC^ *Tnf^–/–^* mice. For panels **E** and **G**, significance was assessed by 2-way ANOVA with Bonferroni’s multiple-comparison test. For panels **F** and **H**, significance was assessed by log-rank Mantel-Cox test. Only significant differences are shown. *P < 0.05; **P < 0.01; ***P < 0.001; ****P < 0.0001. Scale bars: 50 μm. Data represent at least 2 independent experiments.

*LT*α *blockade combined with partial deletion of MyD88 protects against TNF-independent death in A20/Abin-1*^T-ΔIEC^ *Tnf^–/–^ mice*. To determine whether LTα_3_ contributes to IEC injury in vivo, we first performed chromogenic RNA in situ hybridization (RNA-ISH) for *Lta* in the intestine after IEC deletion of *A20* and *Abin-1*. The *A20/Abin-1*^T-ΔIEC^ *Tnf^–/–^* mice exhibited increased *Lta*-positive cells in both the small intestine and colon as compared with *A20^fl/fl^* *Abin-1^fl/fl^* *Tnf^–/–^* mice, correlating increased local *Lta*-expressing cells with histologic severity upon deletion of *A20* and *Abin-1* in IECs (Figure 7, A and B). Similarly, qPCR analysis of the small intestine and colon demonstrated increased *Lta* mRNA in *A20/Abin-1*^T-ΔIEC^ *Tnf^–/–^* mice as compared with *A20^fl/fl^* *Abin-1^fl/fl^* *Tnf^–/–^*, *A20/Abin-1*^T-ΔIEC^ *Tnf^–/–^* *MyD88^–/–^*, and germ-free *A20/Abin-1*^T-ΔIEC^ *Tnf^–/–^* mice (Figure 7C). *Il1b* mRNA exhibited a similar pattern to that of *Lta* (Figure 7D). The increased *Lta* and *Il1b* mRNA by RNA-ISH and qPCR paralleled the pattern of intestinal injury we observed in mice with these genotypes.

To further understand whether blocking LTα provided a survival benefit in these mice, we administered a monoclonal blocking antibody against LTα to *A20/Abin-1*^T-ΔIEC^ *Tnf^–/–^* *MyD88^+/+^* mice and observed a very small but statistically significant increase in weight and median survival as compared with isotype control, with survival increasing from 4.5 to 5.0 days (Figure 7, E and F). As expected, isolated blockade of LTα, without also neutralizing or deleting TNF or MyD88, was insufficient to protect *A20/Abin-1*^T-ΔIEC^ mice (Supplemental Figure 5, A and B). Since both *Lta* and *Il1b* transcripts were elevated in *A20/Abin-1*^T-ΔIEC^ *Tnf^–/–^* intestine (Figure 7, C and D), and since both IL-1β and microbial ligands signal through MyD88, we inhibited both LTα and MyD88 by administering anti-LTα monoclonal antibody to *MyD88^+/–^* heterozygous *A20/Abin-1*^T-ΔIEC^ *Tnf^–/–^* mice. LTα blockade in *MyD88* heterozygous mice reduced weight loss and increased median survival from 4.0 to 9.0 days (Figure 7, G and H). Blocking LTα in vivo reduced weight loss and improved survival, particularly when combined with partial inhibition of MyD88 signaling, supporting a model where LTα and MyD88 agonists contribute to TNF-independent intestinal injury in A20- and ABIN-1–deficient intestinal epithelium.

### LTA and IL1B are relatively increased, and A20 and ABIN-1 protein levels are relatively decreased, in inflamed colon biopsies from patients with IBD.

To determine whether these inflammatory pathways could contribute to intestinal injury in patients with IBD, we measured A20 and ABIN-1 mRNA and protein levels in colonic mucosal biopsies from non-IBD and IBD patients, stratifying the IBD samples by whether they were obtained from endoscopically inflamed or noninflamed areas (Supplemental Table 1). *A20* (*TNFAIP3*) is a TNF- and NF-κB–inducible gene, and mRNA levels were predictably increased in inflamed areas of IBD patients as compared with noninflamed IBD biopsies and non-IBD controls (Supplemental Figure 6A). *ABIN-1* (*TNIP1*) mRNA levels were not significantly different among these groups (Supplemental Figure 6A). However, A20 and ABIN-1 undergo posttranslational regulation and degradation in response to a variety of inflammatory stimuli (72–74), and A20 has been reported to be decreased at the protein level in the mucosa of IBD patients (75). Immunoblot analysis of a subset of mucosal biopsies demonstrated a trend toward lower A20 protein levels in inflamed biopsies and an inverse correlation between A20 mRNA and protein levels (Supplemental Figure 6, B–D). ABIN-1 protein levels were significantly reduced in areas of inflammation in IBD patients (Supplemental Figure 6, B, E, and F). These data suggest that A20 and ABIN-1 proteins are relatively decreased in the intestinal mucosa of IBD patients with active inflammation. Decreased A20 and ABIN-1 levels sensitize IECs to TNFR1-induced apoptosis and necroptosis, so these data suggest that the inflamed mucosa in IBD patients is more susceptible to TNF-dependent and TNF-independent cytotoxic factors.

**Figure 8:**
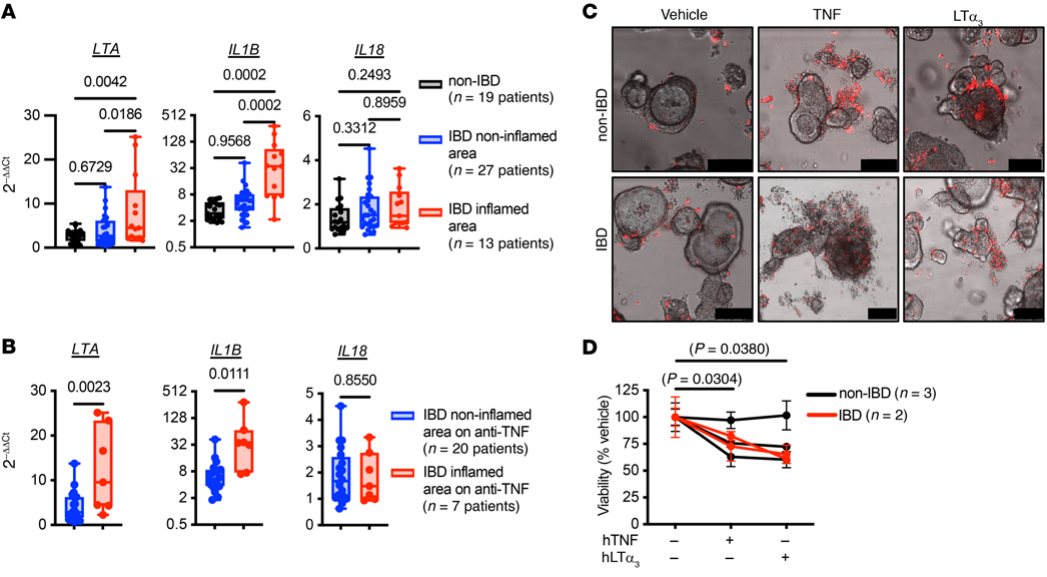
*LTA* and *IL1B* mRNA are increased in inflamed intestinal mucosa from patients with IBD and both TNF and LTα_3_ are cytotoxic to human colonoids. (**A**) qPCR for the indicated mRNA transcripts in colonic mucosal biopsies of inflamed (red) or noninflamed (blue) areas from patients with IBD or without IBD (non-IBD, black). Biopsy samples were obtained at 1 or 2 locations and processed separately; each data point represents 1 patient (median and interquartile ranges, *IL1B* shown with log_2_ *y* axis to display full range). If the colon was entirely inflamed or noninflamed, values from the 2 sets of biopsies were averaged. If biopsies were obtained separately from inflamed and noninflamed colon, then they were processed and displayed separately. (**B**) qPCR for the indicated mRNA transcripts in colonic mucosal biopsies in the subset of patients from **A** on anti-TNF therapy at the time of biopsy (median and interquartile ranges, *IL1B* shown with log_2_ *y* axis to display full range). (**C**) Representative microscopy images of SYTOX dead cell nucleic acid stain (pseudocolor red). Scale bars: 100 μm. (**D**) Quantitative luminescent cell viability assay of human colonoids derived from non-IBD and IBD patients, treated with indicated stimuli for 48 hours (mean ± SEM; 50 ng/mL recombinant human LTα_3_ [hLTα_3_], 25 ng/mL hTNF). For panel **A**, significance was assessed using 1-way ANOVA with Tukey’s multiple-comparison test. For panel **B**, significance was assessed by unpaired Student’s *t* test. For panel **D**, significance was assessed using repeated measures 1-way ANOVA with Dunnett’s multiple-comparison test relative to vehicle.

To further determine whether lymphotoxin or IL-1β contributes to intestinal injury in patients with IBD, we measured *LTA* and *IL1B* in colonic mucosal biopsies from non-IBD and IBD patients, again stratifying by whether they were obtained from endoscopically inflamed or noninflamed areas. *LTA* and *IL1B* were elevated in areas of inflammation (Figure 8A). Another IL-1 family member, *IL18*, was not elevated in inflamed tissue (Figure 8A). These trends were confirmed in a recent meta-analysis (76). Interestingly, in a subset of patients on anti-TNF therapy, both *LTA* and *IL1B* were elevated in areas of active inflammation (Figure 8B), suggesting that local LTα and IL-1β contribute to TNF-independent intestinal injury even when TNF is functionally neutralized. Finally, TNF and LTα_3_ exhibited cytotoxicity in primary human colonoid cultures derived from both non-IBD and IBD patients measured qualitatively by dead cell nucleic acid stain (Figure 8C) and quantitatively using ATP-based luminescent cell viability (Figure 8D). Notably, the human IEC colonoid cytotoxicity induced by both TNF and LTα_3_ was observed in the absence of any death-sensitizing agents (e.g., cycloheximide or second mitochondria-derived activator of caspases [SMAC] mimetics).

## Discussion

Genome-wide association studies have implicated hundreds of genes in CD and UC pathogenesis, but it is unclear how these genes increase disease susceptibility or influence treatment response (77). Since IBD is primarily a complex polygenic disease, except for rare monogenic causes, it follows that model systems often incorporate multiple genes simultaneously to decipher these complex epistatic genetic interactions. While we previously showed that A20 and ABIN-1 cooperatively restrict TNF-dependent IEC injury, this study demonstrates that A20 and ABIN-1 preserve intestinal homeostasis and IEC survival by restricting TNF-independent inflammatory injury. Microbial signals, MyD88 activation, and LTα all contribute to TNF-independent intestinal inflammation in the setting of acute A20 and ABIN-1 deletion in IECs. IEC death induced by A20 and ABIN-1 deficiency can also be blocked by simultaneous inhibition of CASP8-dependent apoptosis and RIPK3-dependent necroptosis. Given that A20 and ABIN-1 restrict both TNF-dependent and TNF-independent IEC injury, this study further reveals why these proteins have such potent antiinflammatory functions in the intestine. Although many previous studies focus on the role of A20 and ABIN-1 in hematopoietic cells, this study and others add to our understanding of the important role these proteins play in nonhematopoietic cells to preserve tissue integrity (9, 16, 37, 39).

Combined acute deletion of A20 and ABIN-1 in the intestinal epithelium is notable in both severity and TNF independence when compared with deletion of other genes in IECs. For example, the fatal enteritis and colitis that develop after simultaneous deletion of A20 and ABIN-1 in IECs rivals the severity reported for intestinal epithelial deletion of NEMO (44, 78), FADD (45, 79), CASP8 (45, 71), RIPK1 (42, 80), ATG16L1 (8), or combined deletion of XBP1 and ATG16L1 (7). Acute deletion of SETDB1 (81) in IECs induces mouse lethality with similar kinetics to the combined deletion of A20 and ABIN-1, although *Setdb1*^–/–^ IECs die primarily from necroptotic cell death. The TNF-independent IEC death observed in *A20/Abin-1*^T-ΔIEC^ *Tnf^–/–^* mice is also unusual when compared with other models. The colitis in mice with conditional IEC knockout of NEMO, FADD, CASP8, and RIPK1 mice is largely reversed by TNF or TNFR1 deletion (42, 44, 45, 78–80). Similarly, the ileitis induced after knockout of XBP1 in IECs is rescued by TNFR1 deficiency (7). Among these models, deletion of A20 and ABIN-1 is unique in that TNF deletion does not confer any significant survival benefit, and it does not appreciably reduce intestinal injury.

The ileal enteropathy associated with IEC knockout of NEMO, FADD, or CASP8 is TNFR1 independent, even though colitis in those mice is TNFR1 dependent. Those models provide some perspective on the TNF-independent IEC death observed in *A20/Abin-1*^T-ΔIEC^ *Tnf^–/–^* mice. Paneth cell loss after IEC knockout of FADD and CASP8 is partially reduced by TNFR1 deletion but is further reduced by deletion of Z-DNA–binding protein 1 (ZBP1), suggesting that ZBP1 contributes to TNFR1-independent death in the ileum (45).

ZBP1 is an IFN-inducible gene product that interacts with RIPK3 via its RHIM domain and activates necroptotic cell death in response to type I and II IFNs (82) and viral nucleic acids (83). It is possible that ZBP1 contributes to TNF-independent IEC death after deletion of A20 and ABIN-1, especially since deletion of TRIF only modestly increased median survival. However, ZBP1 has been shown to activate RIPK3 in IECs when FADD-CASP8 apoptotic death is inhibited (45), or in response to RNA from reactivated endogenous retroviruses after SETDB1 deletion (81). In *A20/Abin-1*^T-ΔIEC^ *Tnf^–/–^* IECs, both FADD and CASP8 are present to activate apoptotic death, and CC3 is detected in the intestinal epithelium and in intestinal organoids, favoring a central role for CASP8-dependent apoptosis rather than potential ZBP1-dependent necroptosis as the dominant TNF-independent death pathway. In *A20/Abin-1*^T-ΔIEC^ *Tnf^–/–^* IECs, RIPK1 is also present and would be predicted to inhibit ZBP1-mediated activation of RIPK3 (84). We cannot entirely exclude a role for ZBP1 in TNF-independent IEC death after deletion of *A20* and *Abin-1*, but the dominant death pathway in *A20/Abin-1*^T-ΔIEC^ *Tnf^–/–^* mice is most consistent with CASP8-mediated apoptosis.

Gasdermin D–mediated (GSDMD-mediated) pyroptotic death is another potential TNF-independent death pathway to consider in A20- and ABIN-1–deficient IECs. A20 restricts NLRP3 inflammasome activation (34, 85), so increased susceptibility to pyroptotic death is a possible sequela of acute simultaneous A20 and ABIN-1 deletion in IECs. Canonical inflammasome activation culminates in cleaved CASP1 and noncanonical inflammasome activation culminates in cleaved CASP11 in mice (86). Both cleaved CASP1 and CASP11 can cleave GSDMD, which in turn causes pyroptotic death (87, 88). CASP1 or -11 cleavage of GSDMD could theoretically precipitate pyroptotic death in *A20/Abin-1*^T-ΔIEC^ *Tnf^–/–^* mice. Combined deletion of RIPK3 and CASP8 completely rescues A20- and ABIN-1–deficient IECs from death, even when CASP1 and -11 are present, but RIPK3 regulates inflammasome activation independently of its role in necroptotic death (34, 89, 90), and CASP8 has been reported to mediate GSDMD cleavage under certain conditions (91, 92). This raises the possibility that combined deletion of RIPK3 and CASP8 rescues *A20/Abin-1*^T-ΔIEC^ mice due to simultaneous blockade of apoptotic, necroptotic, and pyroptotic death. However, GSDMD-dependent death has been reported in IECs primarily when either FADD deficiency or catalytically inactive CASP8 are combined with MLKL deficiency (45, 93, 94). Those recent studies suggest that IECs are driven more toward pyroptotic death when apoptosis and necroptosis are inhibited. A central role for pyroptosis therefore would be less likely in *A20/Abin-1*^T-ΔIEC^ *Tnf^–/–^* mice, where FADD, active CASP8, RIPK1, RIPK3, and MLKL are all present. Taken together, these results suggest that it is possible that some amount of GSDMD-mediated pyroptotic damage occurs in parallel to apoptotic and necroptotic IEC death in *A20/Abin-1*^T-ΔIEC^ *Tnf^–/–^* mice, but apoptotic death is the primary death pathway.

There are multiple potential translational implications for the findings that LTα_3_ and MyD88 contribute to TNF-independent intestinal damage in this model of severe enterocolitis. Deletion of *A20* and *Abin-1* unveils a role for LTα_3_-induced TNF-independent IEC death in primary cells both in vivo and in vitro. To our knowledge, this is the first description of TNF-independent LTα_3_-induced cytotoxicity in IECs in the absence of death-sensitizing agents. There are many potential mechanisms by which patients with IBD may fail to respond to anti-TNF therapy, including activation of alternative inflammatory pathways, neutralizing antibodies, subtherapeutic levels, or inability to block autocrine IEC-derived TNF, but it is tempting to consider whether LTα_3_-mediated TNFR1-induced IEC death could play a role in a subset of patients. Polymorphisms in *LTA* were associated with anti-TNF nonresponse in a small cohort of patients (95), but this was not reproduced in a larger cohort (96). Moreover, etanercept, a soluble TNFR2-Fc receptor that blocks TNF and LTα_3_, was ineffective for CD (97). On the other hand, subsequent data suggest that etanercept is ineffective in part because it does not induce antibody-dependent cell-mediated cytotoxicity or apoptosis of pathogenic TNF-expressing inflammatory cells to the same extent as anti-TNF antibodies (98, 99). Intriguingly, the combination of an anti-TNF monoclonal antibody to neutralize TNF plus etanercept, which additionally neutralizes LTα_3_, was effective in a case report of a patient with severe HLA-B27–associated arthropathy who had failed treatment with either agent alone (100).

The other TNF-independent injury pathway highlighted in this study is MyD88. Microbial signals activating MyD88 in the hematopoietic compartment contribute to intestinal injury, suggesting that investigations into which microbes are most pathogenic in *A20/Abin-1*^T-ΔIEC^ *Tnf^–/–^* gnotobiotic mice would be instructive. Interestingly, the fact that some germ-free *A20/Abin-1*^T-ΔIEC^ *Tnf^–/–^* mice die suggests that sterile inflammation is sufficient to induce death. Dietary or residual bacterial antigens in sterile chow could potentially contribute to intestinal injury in germ-free *A20/Abin-1*^T-ΔIEC^ *Tnf^–/–^* mice (101, 102), but another possibility is that MyD88 activation by endogenous IL-1β and/or IL-18 drives sterile TNF-independent intestinal injury. Both IL-1β and IL-18 have been proposed as potential mediators of anti-TNF nonresponse in patients with IBD (103–106). Furthermore, whether MyD88 activation leads directly to increased LTα_3_ production by innate lymphoid cells or peripheral B cells, for example, remains to be seen (107, 108). Ultimately, having animal models to investigate how MyD88, microbial signals, IL-1, LTα_3_, and various intestinal inflammatory cells cooperatively drive TNF-independent intestinal injury could prove useful in understanding anti-TNF nonresponse in subsets of patients. In addition, TNF- and LTα_3_-induced cytotoxicity in primary human intestinal organoid cultures in the absence of death-sensitizing agents could serve as an in vitro approach to further understand anti-TNF refractory disease.

In summary, we show that microbial signals, MyD88 signaling, and LTα_3_ drive severe TNF-independent enterocolitis after acute deletion of 2 IBD-associated genes, *A20* and *Abin-1*, which can be prevented by combined deletion of *Ripk3* and *Casp8*. This study highlights that A20 and ABIN-1 cooperatively maintain intestinal homeostasis by inhibiting both TNF-dependent and TNF-independent inflammatory intestinal injury. Understanding the genetic determinants of intestinal epithelial health, their epistatic relationships, and how they could influence therapeutic response in models of IBD will provide important mechanistic insights to inform future translational studies.

## Methods

### Mice.

*A20^fl^* and *Abin-1^fl^* mice were generated in the Ma laboratory and were described previously (9, 27, 61, 109). *Tnf^–/–^* mice were purchased from Jackson Laboratories, X. Wang (National Institute of Biological Sciences, Beijing, China) provided *Ripk3^–/–^* mice, and transgenic mice with a tamoxifen-inducible Cre recombinase under the control of the villin promoter (*Vil-cre-ER^T2+^*) mice were a gift from Sylvie Robine (Institut Curie-CNRS; ref. 110). These alleles were backcrossed to *A20^fl^* and *Abin-1^fl^* transgenic mice for more than 8 generations, as previously described (9). Acute deletion of floxed *A20* and *Abin-1* exons in vivo by oral gavage was performed using tamoxifen (2 mg/day; MilliporeSigma, T5648) for 3 consecutive days as indicated for survival analysis. *MyD88^+/–^* and *Trif^+/–^* breeders were maintained on antibiotic water ad libitum with 1 mg/mL ampicillin, 0.5 mg/mL vancomycin, and 1 mg/mL neomycin (all MilliporeSigma), and offspring were transitioned to regular water at weaning. For in vivo monoclonal antibody treatment, mice were injected with 370 μg of antibody on days –2, 0, 1, 2, 5, 7, and 9. For all experimental mice, genotypes were confirmed twice. When possible, littermates were used as controls. Both males and females were included in all experiments, with no observable sex differences. Mice were analyzed between 7 and 12 weeks of age for all experiments. All mice were on the C57BL/6 background.

### Study participants and specimen collection.

Patients undergoing colonoscopy or sigmoidoscopy for standard-of-care indications were screened for study eligibility. All patients gave written informed consent and approval. Cold-forceps biopsy samples were obtained from patients with CD or UC (IBD), and individuals without IBD (non-IBD). Baseline demographic and clinical data for the study participants are provided in Supplemental Table 1. Options were defined by the investigators and participants chose their classifications. Non-IBD patients were patients without known or suspected IBD undergoing elective colonoscopy or sigmoidoscopy for various indications (e.g., colorectal cancer screening). Biopsy samples were categorized as coming from an area that was endoscopically inflamed or noninflamed. Biopsies were obtained from the right (proximal) and/or left (distal) colon. Biopsies were placed in basal organoid media consisting of advanced DMEM/F12 with nonessential amino acids and sodium pyruvate (Thermo Fisher Scientific) supplemented with HEPES (10 mM; Corning), Glutamax (2 mM; Thermo Fisher Scientific), Normocin (100 μg/mL; Invivogen, ant-nr-2), penicillin-streptomycin-neomycin (Thermo Fisher Scientific), and *N*-acetylcysteine (1 mM; MilliporeSigma), supplemented with Y-27632 (10 μM; MedChem Express). Samples were immediately placed on ice and transported to the laboratory for processing.

### Germ-free derivation, maintenance, and conventionalization.

Germ-free *A20/Abin-1*^T-ΔIEC^ *Tnf^–/–^* mice were derived by cesarean delivery into the UCSF Gnotobiotic Core Facility (gnotobiotics.ucsf.edu) and maintained in germ-free isolators. Stool pellets from isolators in the Gnotobiotic Core Facility were screened every 2 to 3 weeks by culture and qPCR, whenever mice were transferred from breeding to experimental isolators, and at the beginning and end of each experiment. 515F and 806R universal primers were used to amplify the V4 region: 515F-GTGCCAGCMGCCGCGGTAA and 806R-GGACTACHVGGGTWTCTAAT. For cecal content conventionalization experiments, cecal contents from SPF mice were resuspended in 10% (w/v) Bacto brain heart infusion (BHI) (VWR) supplemented with 0.05% (w/v) L-cysteine (MilliporeSigma) in aerobic conditions, passed through a 100-μm filter, and 200 μL was administered to germ-free mice by oral gavage 5–7 days prior to tamoxifen treatment.

### CRISPR/Cas9 editing of MyD88, Trif, and Casp8.

Two gRNAs for each target gene were designed by Benchling. The following gRNAs were selected for editing *A20/Abin-1*^T-ΔIEC^ *Tnf^–/–^* mice: *MyD88* guide 1 CCCACGTTAAGCGCGACCAA and *MyD88* guide 2 AAGGAGCTGAAGTCGCGCAT targeting exon 1 of *MyD88*; and *Trif* guide 1 TCTGGAACGCTAATTTCGTG and *Trif* guide 2 CAAGCTATGTAACACACCGC targeting exon 2 of *Trif*. The following gRNAs were selected for editing *A20/Abin-1*^T-ΔIEC^ *Ripk3^–/–^* mice: *Casp8* guide 1 TAGCTTCTGGGCATCCTCGA and *Casp8* guide 2 CTTCCTAGACTGCAACCGAG targeting exon 2 of *Casp8*. The corresponding crRNAs targeting each gene and tracrRNA were annealed and ribonucleoprotein (RNP) complexes were prepared with HiFi Cas9 nuclease ribonucleoprotein (IDT) according to the manufacturer’s instructions. RNPs were electroporated into mouse zygotes as previously described (51), with the main exception that the standard square curve electroporation (2 pulses at 30 V for 3 ms with an interval of 100 ms) was performed once or 4 times with an interval of 3 seconds using a Gene Pulser Xcell (Bio-Rad). Zygotes were cultured to 2-cell embryos and transferred into pseudopregnant females. All mutations were confirmed by cloning and sequencing. Mice with a confirmed deletion or premature stop codon were then backcrossed 3 generations to the parental strains to breed out off-target edits before analysis.

### IEC enteroid culture and confocal microscopy.

For murine enteroid culture, intestinal crypts were isolated from the small intestine as previously described (111), with the modifications of substituting 10% R-spondin-1–conditioned medium for recombinant R-spondin-1, and the addition of Normocin (100 μg/mL, Invivogen). R-spondin-1–expressing 293T cells were a gift from Noah Shroyer (Baylor College of Medicine, Houston, Texas, USA). For all enteroid experiments, enteroids were derived from at least 2 mice on separate occasions and representative data are shown. Deletion of *A20* or *Abin-1* in vitro was performed via treatment with 4-OHT for 24 hours (200 nM; MilliporeSigma). For human colonoid culture, biopsies were cryopreserved, thawed, and digested as previously described (112). Human colonoids were expanded as previously described (113), with WNT surrogate–Fc fusion (ImmunoPrecise) and supplemented with 25% FBS (VWR), CHIR99021 (Cayman Chemical Company), valproic acid (MilliporSigma), [leucine^15^] gastrin-1 human (MilliporeSigma), human R-spondin-3 instead of R-spondin-1 (Peprotech), human stem cell factor (STEMCELL Technologies), human FGF-basic (Peprotech), and human IGF-1 (BioLegend). Confocal imaging of organoids was performed on a Leica SP5 laser scanning confocal system using a 10× dry objective. Images were acquired in a 512 × 512 format, with a line average of at least 3, scan speed of 400 Hz, and pinhole airy unit 1. Excitation for both PI or SYTOX was done with the 488 nm laser line at 30% power with a detection band of 550 to 732 nm (PI) or 520–550 nm (SYTOX). Image analysis was performed using the Leica Application Suite.

### Cell death assays.

Enteroid death assays were performed by resuspending in Matrigel (Corning) and plating 25 μL per well in 96-well flat-bottom opaque plates (Nunc). Deletion of *A20* or *Abin-1* in vitro was performed via treatment with 200 nM 4-OHT for 24 hours, at which point the enteroids were stimulated as indicated. Viability was measured using the CellTiter Glo 3D assay (Promega) according to the manufacturer’s specifications, with the exception that 100 μL of reagent was added to 200 μL of culture for a final volume of 300 μL prior to reading. For colonoids, stimulations were performed as indicated for 48 hours and viability was measured using the CellTiter Glo 3D assay according to the manufacturer’s specifications. Luminescence was read on a SpectraMax M5 (Molecular Devices), analyzed using SoftMax Pro (Molecular Devices), and expressed as percentage viability relative to vehicle control.

### Cell signaling assays and immunoblot analysis.

For enteroid lysates, cultures were resuspended in Cell Recovery Solution (Corning) supplemented with 10 μM Y-27632 (Calbiochem) and incubated for 15 minutes on ice, followed by centrifugation at 500*g* for 5 minutes. Cell pellets were lysed in ice-cold NP40 lysis buffer (1% NP40 [v/v], 50 mM Tris HCl pH 7.4, 150 mM NaCl, and 10% glycerol [v/v]) supplemented with complete EDTA-free Protease Inhibitor Cocktail (Roche), phosphatase inhibitors (1 mM Na_3_VO_4_ and 10 mM NaF), and 10 mM *N*-ethylmaleimide (MilliporeSigma). Colonic mucosal biopsy samples were lysed in Lysing Matrix D tubes (MP Biomedicals) containing RIPA lysis buffer (1% NP40 [v/v], 0.5% sodium deoxycholate [w/v], 0.1% sodium dodecyl sulfate, 50 mM Tris HCl pH 7.4, 150 mM NaCl, and 10% glycerol [v/v]) supplemented with complete EDTA-free Protease Inhibitor Cocktail, phosphatase inhibitors (1 mM Na_3_VO_4_ and 10 mM NaF), 10 mM *N*-ethylmaleimide, and antifoaming Reagent DX (Qiagen). Samples were homogenized using a FastPrep-24 homogenizer (MP Biomedicals). RKO (ATCC, CRL-2577) lysate was used as a reference. Biopsy samples with low lysate protein concentration (below 1000 μg/mL) were excluded. After lysis, samples were centrifuged for 20 minutes at 21,130*g* to remove debris, and the supernatants were quantified using the BCA Protein Assay Kit (Pierce). Lysates were normalized and denatured in LDS Sample Buffer (Invitrogen), followed by resolution in NuPage precast 4% to 12% Bis-Tris gels (Invitrogen) and transferred to PVDF for immunoblotting. Quantitation was performed using Image Lab (Bio-Rad).

### qPCR.

Mouse small intestine or colon (1 cm) was flushed with saline solution and placed in RNAlater (Thermo Fisher Scientific) and stored for 16 to 72 hours at 4°C. For colonic mucosal biopsies, samples were placed in RNAlater and stored for 16 to 72 hours at 4°C. The solution was then aspirated, and samples were stored at –70°C. Samples were then thawed and homogenized in Buffer RLT (Qiagen) supplemented with β-mercaptoethanol using Lysing Matrix D tubes (MP Biomedicals) on the FastPrep-24 (MP Biomedicals), followed by QIAshredder homogenization (Qiagen). RNA was prepared using the RNeasy Mini Kit (Qiagen) with on-column DNase digestion according to the manufacturer’s instructions. Quality was confirmed using the NanoDrop ND-1000 (Thermo Fisher Scientific) and Agilent RNA 6000 Nano Kit on the Agilent 2100 Bioanalyzer, according to manufacturers’ instructions. cDNA was synthesized from 2000 ng of total RNA using the High-Capacity RNA-to-cDNA kit (Thermo Fisher Scientific). qPCR on murine samples was performed using TaqMan probes for mouse *Lta* (Mm00440228_gH), mouse *Il1b* (Mm00434228_m1), and mouse *Actb* (Mm02619580_g1), and the TaqMan Universal Master Mix II with UNG on the QuantStudio 6 (Thermo Fisher Scientific). qPCR on human samples was performed using TaqMan probes for human *LTA* (Hs99999086_m1), *IL1B* (Hs01555410_m1), *IL18* (Hs01038788_m1), *TNFAIP3* (Hs00234713_m1), *TNIP1* (Hs00374581_m1), and *GAPDH* (Thermo Fisher Scientific, 4485713) with the Taqman Multiplex Master Mix (Thermo Fisher Scientific 4461884) on the QuantStudio 6 (Thermo Fisher Scientific). Relative gene abundance was normalized to the mean expression of the housekeeping gene *Actb* or *GAPDH*, and 2^–ΔΔCt^ was calculated relative to a reference sample. All samples were run in duplicate. For mucosal biopsies, if the colon was entirely inflamed or noninflamed, values from the 2 sets of biopsies were averaged. If biopsies were obtained separately from inflamed and noninflamed colon, then they were processed and displayed separately. Samples with low RNA concentration and amplification of GAPDH at Ct greater than 24 were excluded. Data were analyzed using QuantStudio Real-Time PCR Software (Thermo Fisher Scientific).

### Statistics.

Statistical analysis was performed with GraphPad Prism 9. Kaplan-Meier survival curve comparisons were performed using the log-rank Mantel-Cox test. Comparisons between 2 groups were performed by 2-tailed, unpaired Student’s *t* test. Multigroup comparisons were performed by 1-way analysis of variance (ANOVA) if comparing 1 variable per group or 2-way ANOVA if there were multiple variables per group. When comparing every mean to every other mean by ANOVA, Tukey’s multiple comparison test was used. When comparing each mean to a control, Dunnett’s multiple comparison test was used. When comparing only a subset of means, Bonferroni’s multiple-comparison test was used. Categorical variables were analyzed by Fisher’s exact or χ^2^ tests where appropriate. A *P* value of less than 0.05 was used as the threshold for statistical significance.

Histology and immunohistochemistry, antibodies, reagents, and LTα_3_-Fc CM are described in Supplemental Methods.

### Study approval.

All animal studies were conducted in accordance with the UCSF Institutional Animal Care and Use Committee (AN183350). The study was conducted according to Declaration of Helsinki principles and was approved by the Institutional Review Board of UCSF (15-17757 and 19-27302). Written informed consent was received from participants prior to inclusion in the study.

## Author contributions

MGK and A Ma conceived of the project. MGK oversaw the project and designed and performed the experiments with assistance from co–first authors IR and EM. Co–first author order was determined by duration of time spent on the project. IR performed in vivo experiments, in vitro death assays, and helped generate LTα_3_-Fc CM. EM performed enteroid immunoblot analysis and in vitro assays. EM, IR, and MN performed RNA-ISH analysis with advice and guidance from ODK. JLB consented patients and collected and processed patient biospecimens. ZL performed CRISPR/Cas9 zygote editing with guidance from A Marson. JLB, XS, YYR, ZW, RA, and PA assisted with mouse breeding, genotyping, and histology quantitation. KML, PJT, and JAT derived germ-free mice and performed germ-free experiments in the UCSF Gnotobiotics Core Facility. BR provided a key observation that lymphotoxin could contribute to TNF-independent death in this model. BAM, BR, and LS critically reviewed the manuscript and provided helpful discussion. MGK performed the statistical analyses and wrote the manuscript with editing from IR, EM, BAM, A Ma, and input from all co-authors.

## Supplementary Material

Supplemental data

## Version 1 01/25/2022

In-Press Preview

## Version 2 03/01/2022

Electronic publication

## References

[1] Hagiwara C (2002). Increase in colorectal epithelial apoptotic cells in patients with ulcerative colitis ultimately requiring surgery. J Gastroenterol Hepatol.

[2] Zeissig S (2004). Downregulation of epithelial apoptosis and barrier repair in active Crohn’s disease by tumour necrosis factor alpha antibody treatment. Gut.

[3] Kaser A (2010). Inflammatory bowel disease. Ann Rev Immunol.

[4] Goll R, Granlund A van B (2014). Intestinal barrier homeostasis in inflammatory bowel disease. Scand J Gastroenterol.

[5] Hooper LV (2015). Epithelial cell contributions to intestinal immunity. Adv Immunol.

[6] Wlodarska M (2015). An integrative view of microbiome-host interactions in inflammatory bowel diseases. Cell Host Microbe.

[7] Adolph TE (2013). Paneth cells as a site of origin for intestinal inflammation. Nature.

[8] Matsuzawa-Ishimoto Y (2017). Autophagy protein ATG16L1 prevents necroptosis in the intestinal epithelium. J Exp Med.

[9] Kattah MG (2018). A20 and ABIN-1 synergistically preserve intestinal epithelial cell survival. J Exp Med.

[10] Kinchen J (2018). Structural remodeling of the human colonic mesenchyme in inflammatory bowel disease. Cell.

[11] (2019). Colonic epithelial cell diversity in health and inflammatory bowel disease. Nature.

[12] Smillie CS (2019). Intra- and inter-cellular rewiring of the human colon during ulcerative colitis. Cell.

[13] Ma A, Malynn BA (2012). A20: linking a complex regulator of ubiquitylation to immunity and human disease. Nat Rev Immunol.

[14] Jostins L (2012). Host-microbe interactions have shaped the genetic architecture of inflammatory bowel disease. Nature.

[15] Wang S (2013). An enhancer element harboring variants associated with systemic lupus erythematosus engages the TNFAIP3 promoter to influence A20 expression. PLoS Genet.

[16] Catrysse L (2016). A20 prevents chronic liver inflammation and cancer by protecting hepatocytes from death. Cell Death Dis.

[17] Narzo AFD (2016). Blood and intestine eQTLs from an anti-TNF-resistant crohn’s disease cohort inform IBD genetic association loci. Clin Transl Gastroenterol.

[18] Berteau F (2018). Autosomic dominant familial Behçet disease and haploinsufficiency A20: A review of the literature. Autoimmun Rev.

[19] Razani B (2020). Preserving immune homeostasis with A20. Adv Immunol.

[20] Taniguchi K (2021). Novel TNFAIP3 microdeletion in a girl with infantile-onset inflammatory bowel disease complicated by a severe perianal lesion. Hum Genome Var.

[21] Opipari AW (1992). The A20 zinc finger protein protects cells from tumor necrosis factor cytotoxicity. J Biol Chem.

[22] Heyninck K (1999). The zinc finger protein A20 inhibits TNF-induced NF-kappaB-dependent gene expression by interfering with an RIP- or TRAF2-mediated transactivation signal and directly binds to a novel NF-kappaB-inhibiting protein ABIN. J Cell Biol.

[23] Lee EG (2000). Failure to regulate TNF-induced NF-kappa B and cell death responses in A20-deficient mice. Science.

[24] Boone DL (2004). The ubiquitin-modifying enzyme A20 is required for termination of Toll-like receptor responses. Nat Immunol.

[25] Wertz IE (2004). De-ubiquitination and ubiquitin ligase domains of A20 downregulate NF-kappaB signalling. Nature.

[26] Wagner S (2008). Ubiquitin binding mediates the NF-kappaB inhibitory potential of ABIN proteins. Oncogene.

[27] Oshima S (2009). ABIN-1 is a ubiquitin sensor that restricts cell death and sustains embryonic development. Nature.

[28] Skaug B (2011). Direct, noncatalytic mechanism of IKK inhibition by A20. Mol Cell.

[29] Bosanac I (2010). Ubiquitin binding to A20 ZnF4 is required for modulation of NF-κB signaling. Mol Cell.

[30] Nanda SK (2011). Polyubiquitin binding to ABIN1 is required to prevent autoimmunity. J Exp Med.

[31] Tokunaga F (2012). Specific recognition of linear polyubiquitin by A20 zinc finger 7 is involved in NF-κB regulation. EMBO J.

[32] Verhelst K (2012). A20 inhibits LUBAC-mediated NF-κB activation by binding linear polyubiquitin chains via its zinc finger 7. EMBO J.

[33] Lu TT (2013). Dimerization and ubiquitin mediated recruitment of A20, a complex deubiquitinating enzyme. Immunity.

[34] Duong BH (2015). A20 restricts ubiquitination of pro-interleukin-1β protein complexes and suppresses NLRP3 inflammasome activity. Immunity.

[35] Wertz IE (2015). Phosphorylation and linear ubiquitin direct A20 inhibition of inflammation. Nature.

[36] Razani B (2020). Non-catalytic ubiquitin binding by A20 prevents psoriatic arthritis-like disease and inflammation. Nat Immunol.

[37] Vereecke L (2010). Enterocyte-specific A20 deficiency sensitizes to tumor necrosis factor-induced toxicity and experimental colitis. J Exp Med.

[38] Shao L (2013). A20 restricts wnt signaling in intestinal epithelial cells and suppresses colon carcinogenesis. PLoS One.

[39] Vereecke L (2014). A20 controls intestinal homeostasis through cell-specific activities. Nat Commun.

[40] Feoktistova M (2011). cIAPs block ripoptosome formation, a RIP1/caspase-8 containing intracellular cell death complex differentially regulated by cFLIP isoforms. Mol Cell.

[41] Tenev T (2011). The ripoptosome, a signaling platform that assembles in response to genotoxic stress and loss of IAPs. Mol Cell.

[42] Dannappel M (2014). RIPK1 maintains epithelial homeostasis by inhibiting apoptosis and necroptosis. Nature.

[43] Günther C (2015). Caspase-8 controls the gut response to microbial challenges by TNF-α-dependent and independent pathways. Gut.

[44] Vlantis K (2016). NEMO prevents RIP kinase 1-mediated epithelial cell death and chronic intestinal inflammation by NF-κB-dependent and -independent functions. Immunity.

[45] Schwarzer R (2020). FADD and caspase-8 regulate gut homeostasis and inflammation by controlling MLKL- and GSDMD-mediated death of intestinal epithelial cells. Immunity.

[46] Singh S (2018). Systematic review and network meta-analysis: first- and second-line biologic therapies for moderate-severe Crohn’s disease. Aliment Pharmacol Ther.

[47] Feuerstein JD (2020). AGA clinical practice guidelines on the management of moderate to severe ulcerative colitis. Gastroenterology.

[48] Singh S (2020). First- and second-line pharmacotherapies for patients with moderate to severely active ulcerative colitis: an updated network meta-analysis. Clin Gastroenterol Hepatol.

[49] Nguyen NH (2020). Positioning therapies in the management of Crohn’s disease. Clin Gastroenterol Hepatol.

[50] Alsina-Sanchis E (2021). Intraperitoneal oil application causes local inflammation with depletion of resident peritoneal macrophages. Mol Cancer Res.

[51] Chen S (2016). Highly efficient mouse genome editing by CRISPR ribonucleoprotein electroporation of zygotes. J Biol Chem.

[52] Rakoff-Nahoum S (2004). Recognition of commensal microflora by toll-like receptors is required for intestinal homeostasis. Cell.

[53] Asquith MJ (2010). Pathogenic and protective roles of MyD88 in leukocytes and epithelial cells in mouse models of inflammatory bowel disease. Gastroenterology.

[54] Frantz AL (2012). Targeted deletion of MyD88 in intestinal epithelial cells results in compromised antibacterial immunity associated with downregulation of polymeric immunoglobulin receptor, mucin-2, and antibacterial peptides. Mucosal Immunol.

[55] West AP (2006). Recognition and signaling by toll-like receptors. Annu Rev Cell Dev Biol.

[56] Dinarello CA (2009). Immunological and inflammatory functions of the interleukin-1 family. Annu Rev Immunol.

[57] Kaiser WJ, Offermann MK (2005). Apoptosis induced by the toll-like receptor adaptor TRIF is dependent on its receptor interacting protein homotypic interaction motif. J Immunol.

[58] He S (2011). Toll-like receptors activate programmed necrosis in macrophages through a receptor-interacting kinase-3-mediated pathway. Proc Natl Acad Sci U S A.

[59] Kaiser WJ (2013). Toll-like receptor 3-mediated necrosis via TRIF, RIP3, and MLKL. J Biol Chem.

[60] Estornes Y (2012). dsRNA induces apoptosis through an atypical death complex associating TLR3 to caspase-8. Cell Death Differ.

[61] Onizawa M (2015). The ubiquitin-modifying enzyme A20 restricts ubiquitination of the kinase RIPK3 and protects cells from necroptosis. Nat Immunol.

[62] Chaturvedi MM (1994). Tumor necrosis factor and lymphotoxin. Qualitative and quantitative differences in the mediation of early and late cellular response. J Biol Chem.

[63] Bossen C (2006). Interactions of tumor necrosis factor (TNF) and TNF receptor family members in the mouse and human. J Biol Chem.

[64] Etemadi N (2013). Lymphotoxin α induces apoptosis, necroptosis and inflammatory signals with the same potency as tumour necrosis factor. FEBS J.

[65] Chiang EY (2009). Targeted depletion of lymphotoxin-alpha-expressing TH1 and TH17 cells inhibits autoimmune disease. Nat Med.

[66] Dondelinger Y (2013). RIPK3 contributes to TNFR1-mediated RIPK1 kinase-dependent apoptosis in conditions of cIAP1/2 depletion or TAK1 kinase inhibition. Cell Death Differ.

[67] Kondylis V (2017). The interplay of IKK, NF-κB and RIPK1 signaling in the regulation of cell death, tissue homeostasis and inflammation. Immunol Rev.

[68] Takahashi N (2012). Necrostatin-1 analogues: critical issues on the specificity, activity and in vivo use in experimental disease models. Cell Death Dis.

[69] Kaiser WJ (2011). RIP3 mediates the embryonic lethality of caspase-8-deficient mice. Nature.

[70] Oberst A (2011). Catalytic activity of the caspase-8–FLIPL complex inhibits RIPK3-dependent necrosis. Nature.

[71] Günther C (2011). Caspase-8 regulates TNF-α-induced epithelial necroptosis and terminal ileitis. Nature.

[72] Coornaert B (2008). T cell antigen receptor stimulation induces MALT1 paracaspase-mediated cleavage of the NF-kappaB inhibitor A20. Nat Immunol.

[73] Cruz JA (2017). IL-17 signaling triggers degradation of the constitutive NF-κB inhibitor ABIN-1. Immunohorizons.

[74] Chitre AS (2018). A20 upregulation during treated HIV disease is associated with intestinal epithelial cell recovery and function. PLoS Pathog.

[75] Majumdar I (2017). Altered expression of tumor necrosis factor alpha -induced protein 3 correlates with disease severity in ulcerative colitis. Sci Rep.

[76] Linggi B (2021). Meta-analysis of gene expression disease signatures in colonic biopsy tissue from patients with ulcerative colitis. Sci Rep.

[77] Tam V (2019). Benefits and limitations of genome-wide association studies. Nat Rev Genet.

[78] Nenci A (2007). Epithelial NEMO links innate immunity to chronic intestinal inflammation. Nature.

[79] Welz PS (2011). FADD prevents RIP3-mediated epithelial cell necrosis and chronic intestinal inflammation. Nature.

[80] Takahashi N (2014). RIPK1 ensures intestinal homeostasis by protecting the epithelium against apoptosis. Nature.

[81] Wang R (2020). Gut stem cell necroptosis by genome instability triggers bowel inflammation. Nature.

[82] Yang D (2020). ZBP1 mediates interferon-induced necroptosis. Cell Mol Immunol.

[83] Upton JW (2012). DAI/ZBP1/DLM-1 complexes with RIP3 to mediate virus-induced programmed necrosis that is targeted by murine cytomegalovirus vIRA. Cell Host Microbe.

[84] Lin J (2016). RIPK1 counteracts ZBP1-mediated necroptosis to inhibit inflammation. Nature.

[85] Walle LV (2014). Negative regulation of the NLRP3 inflammasome by A20 protects against arthritis. Nature.

[86] Broz P, Dixit VM (2016). Inflammasomes: mechanism of assembly, regulation and signalling. Nat Rev Immunol.

[87] Kayagaki N (2015). Caspase-11 cleaves gasdermin D for non-canonical inflammasome signalling. Nature.

[88] Shi J (2015). Cleavage of GSDMD by inflammatory caspases determines pyroptotic cell death. Nature.

[89] Kang TB (2013). Caspase-8 blocks kinase RIPK3-mediated activation of the NLRP3 inflammasome. Immunity.

[90] Lawlor KE (2015). RIPK3 promotes cell death and NLRP3 inflammasome activation in the absence of MLKL. Nat Commun.

[91] Orning P (2018). Pathogen blockade of TAK1 triggers caspase-8-dependent cleavage of gasdermin D and cell death. Science.

[92] Sarhan J (2018). Caspase-8 induces cleavage of gasdermin D to elicit pyroptosis during Yersinia infection. Proc Natl Acad Sci U S A.

[93] Fritsch M (2019). Caspase-8 is the molecular switch for apoptosis, necroptosis and pyroptosis. Nature.

[94] Newton K (2019). Activity of caspase-8 determines plasticity between cell death pathways. Nature.

[95] Taylor KD (2001). ANCA pattern and LTA haplotype relationship to clinical responses to anti-TNF antibody treatment in Crohn’s disease. Gastroenterology.

[96] Dideberg V (2006). Lymphotoxin alpha gene in Crohn’s disease patients: absence of implication in the response to infliximab in a large cohort study. Pharmacogenet Genomics.

[97] Sandborn WJ (2001). Etanercept for active Crohn’s disease: a randomized, double-blind, placebo-controlled trial. Gastroenterology.

[98] Mitoma H (2008). Mechanisms for cytotoxic effects of anti-tumor necrosis factor agents on transmembrane tumor necrosis factor alpha-expressing cells: comparison among infliximab, etanercept, and adalimumab. Arthritis Rheum.

[99] Mitoma H (2018). Molecular mechanisms of action of anti-TNF-α agents - Comparison among therapeutic TNF-α antagonists. Cytokine.

[100] Sheehy C (2006). Effective co-administration of infliximab and etanercept following the failure of sequential anti-TNF agents in a patient with HLA-B27-associated arthropathy. Rheumatology (Oxford).

[101] Kim KS (2016). Dietary antigens limit mucosal immunity by inducing regulatory T cells in the small intestine. Science.

[102] Schwarzer M (2017). Diet matters: endotoxin in the diet impacts the level of allergic sensitization in germ-free mice. PLoS One.

[103] Bank S (2018). Genetically determined high activity of IL-12 and IL-18 in ulcerative colitis and TLR5 in Crohns disease were associated with non-response to anti-TNF therapy. Pharmacogenomics J.

[104] Martin JC (2019). Single-cell analysis of Crohn’s disease lesions identifies a pathogenic cellular module associated with resistance to anti-TNF therapy. Cell.

[105] Mitsialis V (2020). Single-cell analyses of colon and blood reveal distinct immune cell signatures of ulcerative colitis and Crohn’s disease. Gastroenterology.

[106] Friedrich M (2021). IL-1-driven stromal-neutrophil interactions define a subset of patients with inflammatory bowel disease that does not respond to therapies. Nat Med.

[107] Ansel KM (2000). A chemokine-driven positive feedback loop organizes lymphoid follicles. Nature.

[108] The Immunological Genome Project Consortium (2008). The Immunological Genome Project: networks of gene expression in immune cells. Nat Immunol.

[109] Tavares RM (2010). The ubiquitin modifying enzyme A20 restricts B cell survival and prevents autoimmunity. Immunity.

[110] Marjou FE (2004). Tissue-specific and inducible Cre-mediated recombination in the gut epithelium. Genesis.

[111] Sato T, Clevers H (2013). Primary mouse small intestinal epithelial cell cultures. Methods Mol Biol.

[112] Konnikova L (2018). High-dimensional immune phenotyping and transcriptional analyses reveal robust recovery of viable human immune and epithelial cells from frozen gastrointestinal tissue. Mucosal Immunol.

[113] Miao Y (2020). Next-generation surrogate wnts support organoid growth and deconvolute frizzled pleiotropy in vivo. Cell Stem Cell.

