# Supplementary material for: Microbial signals, MyD88, and lymphotoxin drive TNF-independent intestinal epithelial tissue damage
Source: J Clin Invest. 2022 Mar 1;132(5):e154993. doi: 10.1172/JCI154993 (PMC8884902; doi:10.1172/JCI154993)
Supplement: Supplemental data [file jci-132-154993-s187.pdf]

Histology and Immunohistochemistry

Mice were individually euthanized, dissected, and their resected intestinal tissue was immediately fixed in 10% neutral-buffered formalin (Millipore Sigma) 40h after treatment with tamoxifen. Tissue specimens were fixed for 16-24 hours, washed in PBS three times, then placed in 70% ethanol and shipped to Histowiz (Brooklyn, NY) for paraffin embedding, sectioning, staining, and high-resolution (40X) scanning according to standard protocols. H&E histologic severity quantitation was performed using a scoring system previously described for genetic IBD models affecting the small intestine (score range 0-15) or colon (score range 0-12) (114). For CC3-stained sections (Cell Signaling Technology 9661), 10 consecutive crypts from each of 5 sections for small intestine and colon (50 crypts per mouse per segment) were counted. Chromogenic RNA *in situ hybridization* (RNA-ISH) was performed using RNAscope® Probe Mm-*Lta* (Advanced Cell Diagnostics 317231) and RNAscope 2.5 HD Assay-Brown Kit (Advanced Cell Diagnostics 322370) according to the manufacturer's specifications. Slides were immediately covered with a coverslip using Cytoseal. Slides were then scanned at 40X magnification with a Zeiss Axio ScanZ.1 (Zeiss). DAB-positive areas were identified against a hematoxylin background in all 5 sections of small intestine and colon on each slide using QuPath v0.2.3. At least two and up to three blinded readers assessed histologic disease severity, CC3 quantitation, and *Lta* quantitation and the results were averaged.

Antibodies and reagents

Antibodies directed against A20 (Cell Signaling Technology, 5630), ABIN-1 (Millipore Sigma HPA037893), Caspase 8 (Cell Signaling Technology mouse specific 4927 and 4790), CC8 (Cell Signaling Technology 8592), RIPK1 (Cell Signaling Technology 3493), Caspase 3 (Cell Signaling Technology 9662), CC3 (Cell Signaling Technology 9661), PARP (Cell Signaling Technology 9542), mouse RIPK3 (Cell Signaling Technology 95702), phospho-RIPK3 (T231/S232) (Cell Signaling Technology 91702), phospho-RIPK1 (Ser166) rodent specific (Cell Signaling Technology 31122), and GAPDH (Millipore MAB374), were used for western blot. Anti-CC3 (Cell Signaling Technology 9661) was also used for IHC studies. Mouse IFN-β ELISA (PBL assay bioscience) was used according to the manufacturer's instructions. Recombinant mouse TNF, mouse IL-18, mouse TL1A, mouse $LT\alpha_1\beta_2$, human $LT\alpha_3$, mouse TNFR1-Fc, and mouse LTBR-Fc were purchased from R&D systems. Recombinant mouse RANKL was purchased from BioLegend. Recombinant mouse IL-1β and mouse LIGHT were purchased from Peprotech. Recombinant human TNF was purchased from Enzo Life Sciences.

Pam3CSK4, poly(I:C) HMW, and LPS from E. coli O111:B4 were purchased from Invivogen. Necrostatin-1s (BioVision 2263) and Emricasan (MedChem Express HY-10396) were used at 50μM final concentration. S5H3.2.2 hybridoma cell line (ATCC) expressing monoclonal hamster anti-mouse LTα (65) was provided to BioXcell (Lebanon, New Hampshire) for production of purified monoclonal antibody for in vivo use. InVivoMAb Armenian hamster IgG isotype control anti-glutathione S-transferase (BioXcell #BE0260) was used as a negative control.

$LT\alpha_3$-Fc conditioned media

Codon-optimized gblocks (IDT) encoding mouse LTα (amino acids 1-202; UniProtKB/Swiss-Prot P09225.1) followed by a GSG linker and mouse IgG2a Fc fusion (amino acid 99-330; UniProtKB/Swiss-Prot P01863) were synthesized and then cloned into pENTR (Addgene Plasmid #17398) using InFusion cloning according to manufacturer's instructions (Clontech). The insert sequence was confirmed and then cloned into pLEX_307 (Addgene Plasmid #41392) using the Gateway Cloning LR reaction according to manufacturer's instructions (ThermoFisher Scientific). Lentivirus particles were prepared by co-transfection with the packaging plasmid psPAX2 (Addgene Plasmid #12260) and the envelope plasmid pMD2.G (Addgene Plasmid #12259) into HEK293T (ATCC) cells using Lipofectamine 2000 (ThermoFisher Scientific). The lentivirus-containing medium was collected at 72 h post-transfection. After centrifugation, the lentivirus medium was filtered using a 0.45 μm syringe filter. HEK293T cells were transduced with diluted lentivirus and selected with puromycin 5 ug/ml for several passages. The puromycin was removed and mLTα-Fc conditioned media (mLTα-Fc CM) was collected and filtered over 0.22μm filter. The mLTα-Fc CM was quantitated by ELISA with goat polyclonal LTα capture antibody (R&D systems AF749), rat monoclonal LTα detection antibody (R&D systems MAB749) biotinylated with ChromaLink biotin according to manufacturer's instructions (Trilink Biotechnologies), a recombinant mouse lymphotoxin alpha ELISA reference (R&D systems), peroxidase streptavidin (Jackson ImmunoResearch), and 1-Step Ultra TMB-ELISA (ThermoFisher Scientific).

References:

114. Erben U et al. A guide to histomorphological evaluation of intestinal inflammation in mouse models. *International journal of clinical and experimental pathology* 2014; 7(8):4557-4576.

Supplemental Figure 1

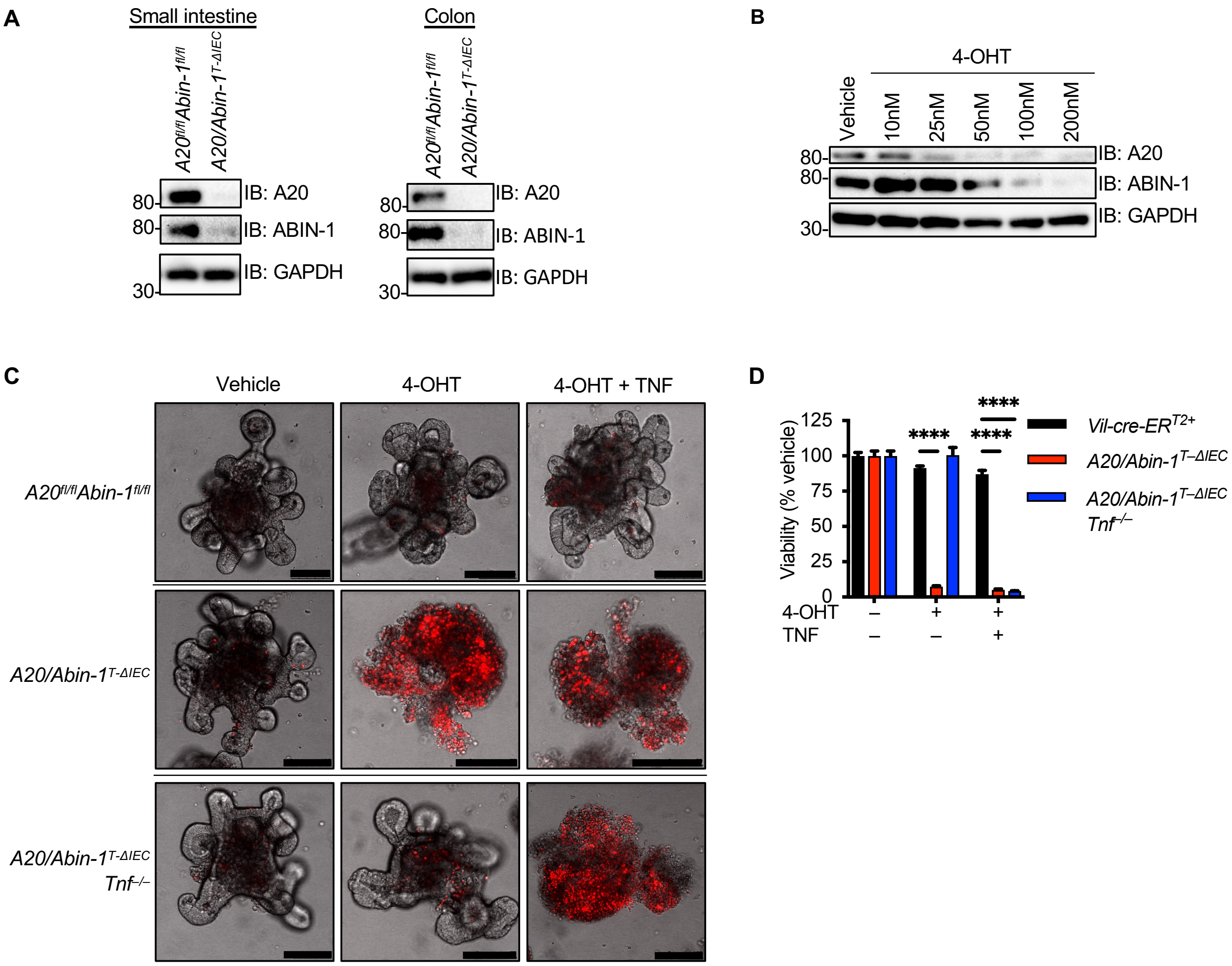

Supplemental Figure 1. Tamoxifen-induced deletion of floxed *A20* and *Abin-1* on a *Vil-cre-ER*$^{T2+}$ background in vivo and in vitro. (**A**) Immunoblotting analysis of IECs with the indicated genotypes 40h after treatment with 2mg tamoxifen per os (p.o.). (**B**) Immunoblotting analysis of *A20/Abin-1*$^{T\text{-}\Delta IEC}$*Tnf*$^{-/-}$ enteroid cultures treated with the indicated concentration of 4-OHT for 24h. For panels (**A,B**), lysates were immunoblotted with the antibodies indicated on the right. (**C**) Representative confocal microscopy images of PI-stained enteroids (pseudocolor red) from indicated genotypes of mice treated with vehicle or 4-OHT for 24h followed by 24 h of 2.5 ng/ml TNF as indicated. Scale bars, 100μm. (**D**) Quantitative luminescent cell viability assay of enteroid cultures treated as described in (**C**) (mean ± SEM). For panel (**D**), significance was assessed by two-way ANOVA with Dunnett's multiple comparison test comparing *A20/Abin-1*$^{T\text{-}\Delta IEC}$ and *A20/Abin-1*$^{T\text{-}\Delta IEC}$*Tnf*$^{-/-}$ to control *Vil-cre-ER*$^{T2+}$ enteroids.

Supplemental Figure 2

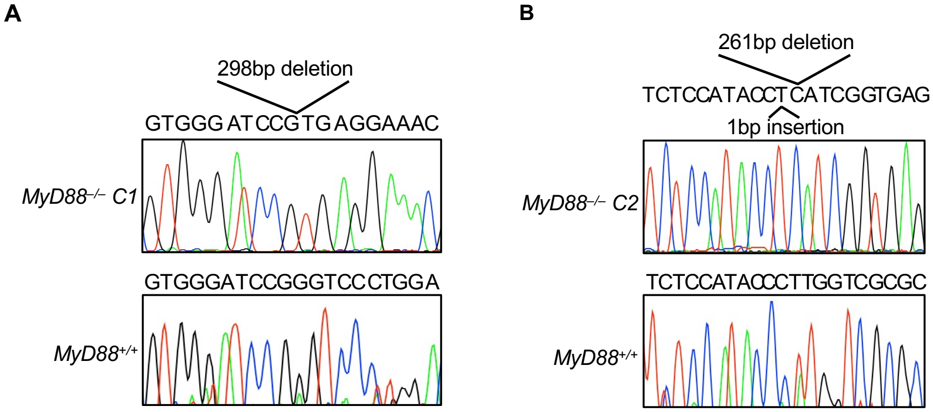

Supplemental Figure 2. Editing of *MyD88* in *A20/Abin-1$^{T\text{-}\Delta IEC}$Tnf$^{-/-}$* mice. Nucleotide chromatogram and sequence of the *MyD88* PCR amplicon surrounding the CRISPR-Cas9-targeted site for (**A**) *MyD88 C1* and (**B**) *MyD88 C2* in *A20/Abin-1$^{T\text{-}\Delta IEC}$Tnf$^{-/-}$* mice.

Supplemental Figure 3

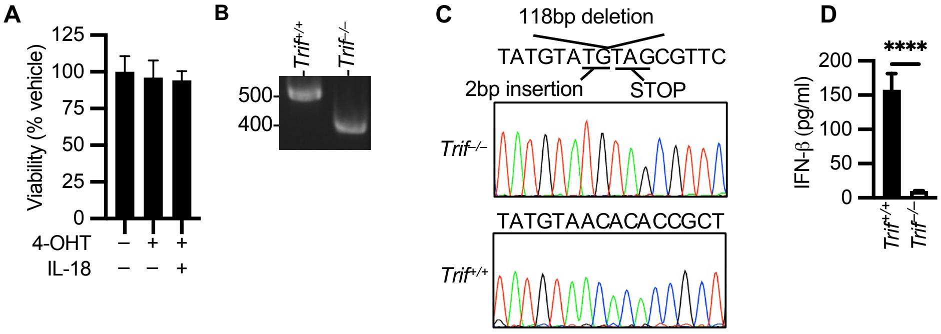

Supplemental Figure 3. IL18 stimulation of *A20/Abin-1*$^{T\text{-}\Delta IEC}$*Tnf*$^{-/-}$ enteroids and editing of *Trif* in *A20/Abin-1*$^{T\text{-}\Delta IEC}$*Tnf*$^{-/-}$ mice. (**A**) Quantitative luminescent cell viability assay of *A20/Abin-1*$^{T\text{-}\Delta IEC}$*Tnf*$^{-/-}$ enteroid cultures treated with the indicated stimuli (mean ± SEM; IL-18 25ng/ml). (**B**) Agarose gel electrophoresis and (**C**) nucleotide chromatogram and sequence of the *Trif* PCR amplicon surrounding the CRISPR-Cas9-targeted site in *Trif* for *A20/Abin-1*$^{T\text{-}\Delta IEC}$*Tnf*$^{-/-}$ mice. (**D**) IFN-β ELISA of mouse splenocytes with the indicated genotype stimulated with 100 µg/ml poly(I:C). For panel (**A**) significance was assessed using one-way ANOVA with Dunnett's multiple comparison test relative to vehicle alone. For panel (**D**), significance was assessed using unpaired t-test. Only significant differences are shown; ****=$p < 0.0001$. Data represent at least two independent experiments.

Supplemental Figure 4

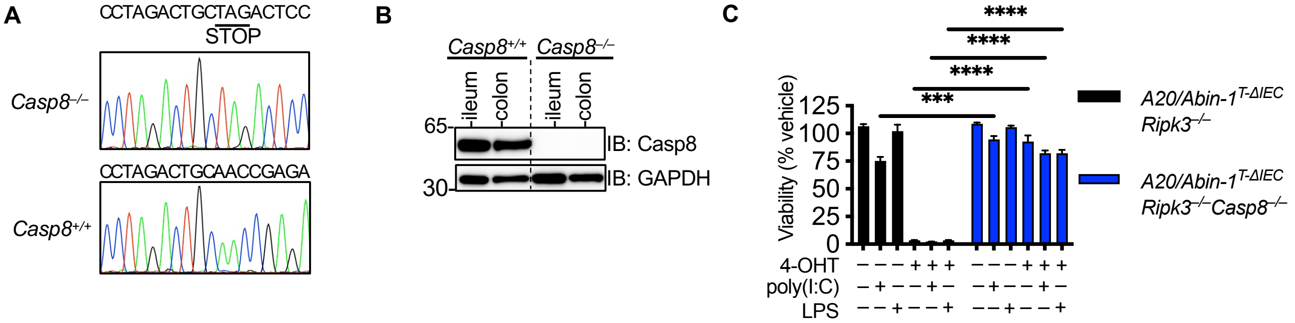

Supplemental Figure 4. Deletion of *Casp8* in *A20/Abin-1$^{T\text{-}\Delta IEC}$Ripk3$^{-/-}$* mice and poly(I:C) stimulation of *A20/Abin-1$^{T\text{-}\Delta IEC}$Ripk3$^{-/-}$* and *A20/Abin-1$^{T\text{-}\Delta IEC}$Ripk3$^{-/-}$Casp8$^{-/-}$* enteroids. (**A**) Nucleotide chromatogram and sequence of the *Casp8* PCR amplicon surrounding the CRISPR-Cas9-targeted site in *Casp8* for *A20/Abin-1$^{T\text{-}\Delta IEC}$Ripk3$^{-/-}$* mice. (**B**) Immunoblot of intestinal epithelial cells from *A20/Abin-1$^{T\text{-}\Delta IEC}$Ripk3$^{-/-}$* mice with the indicated *Casp8* genotypes. (**C**) Quantitative luminescent cell viability assay of enteroids with the indicated genotype treated with indicated stimuli, (mean ± SEM). For panel (**C**) statistical significance was assessed by two-way ANOVA with Bonferroni's multiple comparison test between genotypes for each stimulation condition. Only significant differences are shown; ***=p < 0.001; ****=p< 0.0001. Data represent at least two independent experiments.

Supplemental Figure 5

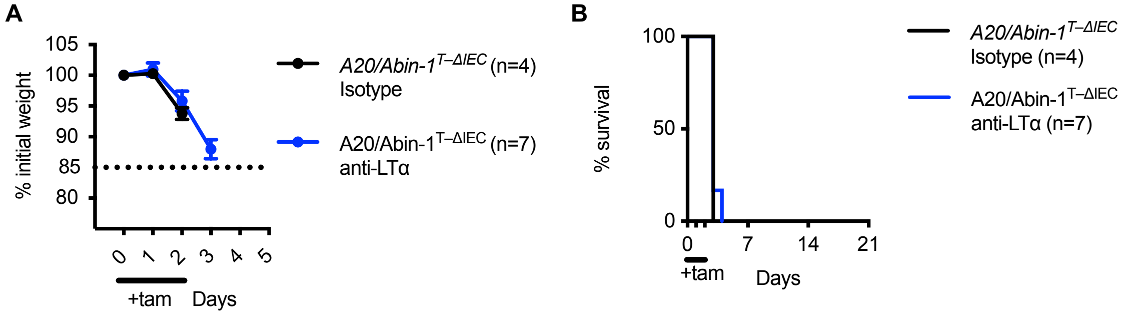

Supplemental Figure 5. Anti-LTα alone, in the absence of TNF neutralization or depletion, does not improve survival in *A20/Abin-1$^{T-\Delta IEC}$* mice. (**A**) Weight curve and (**B**) Kaplan-Meier survival curves of the indicated genotypes of tamoxifen-treated mice treated with anti-LTα or isotype control. For panel (**A**), significance was assessed by two-way ANOVA with Bonferroni's multiple comparison test. For panel (**B**), significance was assessed by Log-rank Mantel-Cox test. Only significant differences are shown.

Supplemental Figure 6

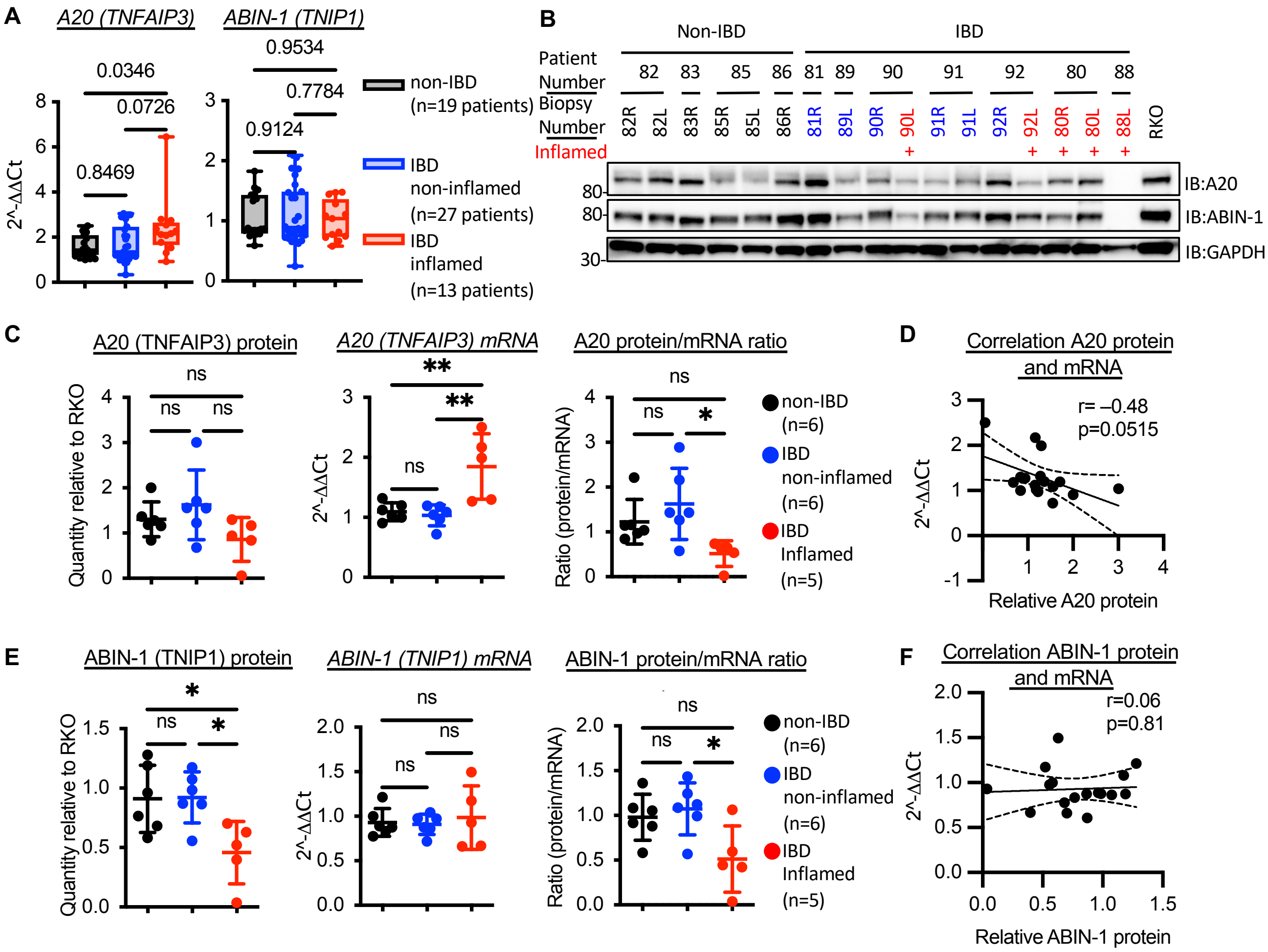

Supplemental Figure 6. A20 and ABIN-1 protein levels are relatively decreased in inflamed intestinal mucosa in patients with IBD. (**A**) qPCR for the indicated mRNA transcripts in colonic mucosal biopsies (median and interquartile ranges) and (**B**) immunoblot of lysates from a subset of biopsies from inflamed (red) and/or non-inflamed (blue) areas from patients with IBD or without IBD (non-IBD, black). Patient and biopsy number are shown, along with location (R-right colon; L-left colon). Biopsy samples were obtained at one or two locations and processed separately, Each data point represents one patient. RKO cell line mRNA and lysate were used as a reference. (Median and interquartile ranges). (**C**) Relative A20 (TNFAIP3) protein (left panel), mRNA (middle panel), and protein/mRNA (right panel) quantitation from a subset of patients in (**A**) (mean ± SD). (**D**) Correlation between A20 (TNFAIP3) protein and mRNA in mucosal biopsies. (**E**) Relative ABIN-1 (TNIP1) protein (left panel), mRNA (middle panel), and protein/mRNA (right panel) quantitation from a subset of patients in (**A**) (mean ± SD). (**F**) Correlation between ABIN-1 (TNIP1) protein and mRNA in mucosal biopsies. For panels (**A, C, E**), significance was assessed using one-way ANOVA with Tukey's multiple comparison test. For panels (**D**,**F**), pearson correlation coefficient and corresponding p-value were calculated. ns=not significant, *=p<0.05, **=p<0.01. Data represent two independent measurements.

Characteristic			Non-IBD (n=19)	IBD (n=33)	p-value
Gender (F/M)			11/8	13/20	0.25
Age, years median (IQR1–IQR3)			53 (36-61)	37 (30-48)	0.005
Disease type n (%)					<0.0001
	UC		0	20 (61%)	
	CD		0	13 (39%)	
	non-IBD		19 (100%)	0	
Racial and Ethnic Category n (%)					0.61
	White		15 (79%)	28 (85%)	
	Hispanic		1 (5%)	0	
	Black		2 (11%)	2 (6%)	
	Asian		1 (5%)	2 (6%)	
	Other/unknown		0	1 (3%)	
Medication					<0.0001
	Steroids n (%)		0	1 (3%)	
	Mesalamine n (%)		0	6 (18%)	
	Immunomodulator n (%)		1 (6%)	13 (39%)	
	Biologic n (%)		0	25 (76%)	
		anti-TNF		23 (70%)	
		other biologic		2 (6%)	
Endoscopic inflammation n (%)			0	13 (39%)	0.0018

Supplemental Table 1. Baseline demographic and clinical data for study participants. Categorical variables were analyzed by Fisher's exact or Chi-square tests where appropriate, and non-normally distributed continuous variables were compared using Mann-Whitney test.
